# No systematic difference in obesity susceptibility between two groups of zoo primates with distinct digestive physiologies: hindgut-fermenting lemurs and foregut-fermenting colobines

**DOI:** 10.1007/s10329-026-01245-4

**Published:** 2026-02-21

**Authors:** Christiane Krauss, Elisa Garand, Max Hahn-Klimroth, Dennis W. H. Müller, Paul W. Dierkes, Ikki Matsuda, Marcus Clauss, João Pedro Meireles

**Affiliations:** 1 Clinic for Zoo Animals, Exotic Pets and Wildlife, Vetsuisse Faculty, University of Zurich, Winterthurerstr. 260, 8057 Zurich, Switzerland; 2 Bioscience Education and Zoo Biology, Goethe-University of Frankfurt am Main, Max-von-Laue-Straße 13, 60438 Frankfurt am Main, Germany; 3 Zoological Garden Halle (Saale), Fasanenstr. 5a, Saale, 06114 Halle, Germany; 4 Wildlife Research Center, Kyoto University, Sakyo, Kyoto, 606-8203 Japan; 5 Chubu Institute for Advanced Studies, Chubu University, Kasugai, Aichi 487‑8501 Japan; 6 Institute for Tropical Biology and Conservation, Universiti Malaysia Sabah, Jalan UMS, 88400 Kota Kinabalu , Sabah Malaysia

**Keywords:** Primates, Colobinae, Lemuridae, Diet, Nutrition, Free-ranging, Ex situ

## Abstract

Obesity is an often-stated issue in zoo animals, including primates, and has historically been particularly emphasized for lemurs. By contrast, similar reports for colobine species—which are literally called ‘slim monkeys’ in at least one other language—are absent. This has been hypothsized to be linked to the colobine foregut fermentation system; diets high in easily digestible carbohydrates might disturb the microbiome in the foregut, similar to acidosis in domestic ruminants, leading to reduced intake and disease rather than obesity, but empirical data is lacking. We compared body mass (BM) data for 14 lemur species (7187 individuals) and 10 colobine species (1443 individuals) with literature data on free-ranging specimens to test this concept. In contrast to expectations, a similar percentage of species had a majority of individuals above the ‘healthy’ BM range in at least one sex (36% of lemur and 40% of colobine species). However, colobines had a higher percentage of individuals considered ‘underweight’, especially in the two rarely kept species with the more complex ‘quadripartite’ stomachs (*Nasalis larvatus*, *Pygathrix nemaeus*). Neither sample size, sexual size dimorphism, the degree of folivory, nor indications for a seasonal physiology were clearly associated with species’ propensity for zoo obesity. There were no clear historical trends in the development of obesity in these species. We conclude that no general rules about species’ susceptibility to obesity appear to apply. Body mass and condition of zoo primates should be monitored continuously, and feeding regimes possibly be adapted to contain diets of higher fibre levels more similar to those reported in natural diets.

**Supplementary Information:**

The online version contains supplementary material available at https://doi.org/10.1007/s10329-026-01245-4.

## Introduction

Primates exhibit remarkable diversity in terms of natural diet and digestive physiology, reflecting their ecological adaptations and evolutionary histories (Lambert 1998; Hohmann et al. 2006; Clauss et al. 2008). Among them, broadly speaking, lemurs and colobines present two contrasting models of feeding ecology and digestive physiology. Lemurs, a basal primate lineage isolated on Madagascar, have evolved under conditions of seasonal resource scarcity and high ecological unpredictability and seasonality (Fietz and Ganzhorn 1999; Norscia et al. 2006; Donati et al. 2007). As a result, their diets are notably flexible, ranging from frugivory and folivory to opportunistic feeding on flowers, insects, and even tree bark, depending on species and habitat (LaFleur and Sauther 2015; Beeby and Baden 2021; Mihaminekena et al. 2024). Lemurs are ‘hindgut fermenters’ with a variable caecum and colon morphology (Campbell et al. 2000) and corresponding variation in digesta passage and digestive efficiency (Campbell et al. 2004a, b). In contrast, langurs or colobine monkeys are distributed across Central Africa, South and Southeast Asia, are highly adapted folivores (Matsuda and Clauss 2022). Their specialised foregut fermentation system enables efficient digestion of fibrous plant material (Amato et al. 2016; Matsuda et al. 2017; Matsuda and Clauss 2022), and while fruits, seeds, and flowers may supplement their diets, leaves dominate their nutritional intake (Amato et al. 2016; Matsuda et al. 2020, 2022a).

Comparing these two primate groups provides insights into feeding strategies, nutritional ecology and digestive physiology. Such comparisons become particularly relevant in zoo settings, where diet and environment are managed by humans. The degree to which primates can adapt to non-native diets has direct consequences for both animal health and husbandry practices. Zoos frequently replace natural diets with cultivated fruits and commercial primate pellets, which are typically higher in sugar and starch, and lower in fibre, than wild foods, and are offered consistently year-round rather than seasonally (Nijboer et al. 2006b; Schwitzer 2009; Donadeo et al. 2016; Caravaggi et al. 2018). Direct comparisons between diets fed in zoos to those in natural habitats in gentle lemurs (*Hapalemur* spp.) (Feistner and Mutschler 1999), black lemurs (*Eulemur flavifrons*) (Polowinsky and Schwitzer 2009) and red-shanked douc langurs (*Pygathrix nemaeus*) (Schwitzer et al. 2006a), as well as faecal nutrient levels in free-ranging and captive proboscis monkeys (*Nasalis larvatus*) (Matsuda et al. 2018), suggest that zoo diets are lower in fibre and higher in easily digestible carbohydrates. It has been hypothesised that such diets affect foregut and hindgut fermenters differently (Clauss and Dierenfeld 2008).

Lemurs are generally easier to feed in captivity, as many species are ecological generalists capable of utilising a wide range of foods (LaFleur and Sauther 2015; Irwin et al. 2025). Additionally, their hindgut fermentation system can handle low-fibre, high-sugar diets more easily: as the ingested food first passes through the monogastric stomach and small intestine, highly digestible carbohydrates are simply digested and absorbed before they can reach the sites of microbial fermentation in the hindgut. In theory, therefore, lemurs should be more susceptible to obesity than malfermentation (Clauss and Dierenfeld 2008).

In contrast, colobines have long posed challenges in zoo management—putatively because of the combination of their predominantly folivorous, browse-based diets and their foregut fermentation strategy (Matsuda and Clauss 2022). A browse-based diet is difficult to replicate in zoo settings, particularly in the temperate zone with deciduous forests, outside the animals’ native ranges, where suitable browse may be scarce or seasonally unavailable (Agoramoorthy et al. 2004). The specialised foregut-fermenting digestive system possibly constrains dietary substitution, as provision of diets low in fibre (or high in easily digestible carbohydrates) has been associated with malfermentation in the forestomach; this is because these easily digestible carbohydrates will reach the site of microbial fermentation directly in a foregut system (Clauss and Dierenfeld 2008). Associated problems are weight loss, softer faeces, and digestive upsets such as diarrhoea (Nijboer and Dierenfeld 1996; Edwards and Ullrey 1999; Nijboer et al. 2006a; Hoshino et al. 2023), ultimately linked to a reduced longevity (Osman Hill 1964; Hollihn 1973; Collins and Roberts 1978). A German word for colobine monkeys is ‘Schlankaffen’ (Kuhn 1964), i.e. ‘the slim monkeys’, suggesting that these animals rarely have an obese body shape. This difference between hindgut-fermenting primates on the one hand, including lemurs, and the foregut-fermenting colobine monkeys on the other, is apparently supported by the fact that the latter appear as the primate group least susceptible to obesity in the comparative analyses by Leigh (1994) and Pontzer (2023). However, in parallel to other foregut fermenters in which obesity has been reported (e.g., macropods, Dunham et al. 2022; or hippos, Meireles et al. 2025), if easily fermentable nutrients are provided only at a certain but not excessive rate, obesity might be expected in colobines as well.

Under conditions of high constant availability of energy-dense food items, obesity is a looming problem. This is evident in the global human population, where obesity has been on the rise for a considerable time, regardless of a nation’s level of development (Phelps et al. 2024). It has also been described as an important characteristic of certain zoo animal populations (Garand et al. 2024; Meireles et al. 2025). Different comparisons were also published on individual primate taxa, such as gorillas (*Gorilla gorilla*) (Cousins 1972), vervet monkeys (*Chlorocebus aethiops sabaeus*) (Turner et al. 2016) or chimpanzees (*Pan troglodytes*) (Curry et al. 2023). But ever since the observations of obesity in captive mongoose lemurs (*Lemur mongoz*) by Schaaf and Stuart (1983) and additionally in *Lemur catta* and *Eulemur fulvus* by Pereira and Pond (1995), the susceptibility of lemurs to obesity has received much attention, as in the more systematic studies on *Varecia variegata* by Schwitzer and Kaumanns (2001; 2009) and several taxon-wide assessments (Terranova and Coffman 1997; Goodchild and Schwitzer 2008; Taylor et al. 2012; Mellor et al. 2020), making this group potentially the best-studied primate taxon in this respect. Thus, several lemur species have been identified as being prone to obesity in managed care.

The prevalence of overweight in zoo primates has been linked to the provision of energy-dense diets (Goodchild and Schwitzer 2008; Caravaggi et al. 2018; Van Mulders et al. 2024) that often do not align with the diets that the species encounter in the wild, which often are higher in fibre and show distinct seasonal variation. Primates in zoos also spend less time foraging and feeding than they do in the wild (Melfi and Feistner 2002; Schwitzer et al. 2006b; Yamanashi and Hayashi 2011; Bailey et al. 2020; Kamaluddin et al. 2022). For example, colobines such as proboscis monkeys exhibit selective, wide-ranging feeding behaviours that demand significant locomotion, including swimming across rivers daily (Bennett and Sebastian 1988). In captivity, however, behavioural and locomotor patterns can differ dramatically, as seen in *Pygathrix* species, where up to half of locomotor time may be spent arm-swinging rather than travelling (Bailey et al. 2020).

Seasonality can further complicate the picture. Several lemur species show pronounced seasonal fluctuations in reproduction (Heldstab et al. 2021), and therefore also in activity, diet, and body mass. For example, fat-tailed dwarf lemurs (*Cheirogaleus medius)* can double their body mass in as little as two weeks before hibernation in the wild (Fietz and Ganzhorn 1999), though such fluctuations are dampened under captive conditions where true hibernation may not occur (Hladik et al. 1980). Captive ring-tailed lemur (*Lemur catta)* show a seasonal or photoperiod-dependent variation in intake and body mass (Pereira 1993; Firmino et al. 2020; Rimbach and Pontzer 2023) that corresponds to seasonal differences in adipose tissue accretion in natural habitats (Simmen et al. 2010). Similarly, free-ranging and captive *Propithecus* spp. show seasonal cycles of mass loss and gain (Richard et al. 2000; Lewis and Kappeler 2005; Rimbach and Pontzer 2023)*.* The collared brown lemur* (Eulemur collaris)* exhibits seasonal feeding patterns, with significantly higher diurnal feeding during the hot–wet season compared to other times of year (Donati et al. 2007). Adaptations to accrue body mass as adipose tissue to manage a seasonal environment could, in theory, make a species more susceptible to obesity under conditions of permanent access to high-quality resources. Actually, seasonally fat-accruing lemurs such as grey mouse lemurs (*Microcebus murinus*) have been used in translational research to evaluate pharmacological interventions against obesity (Dal-Pan et al. 2010). The fact that colobine monkeys generally do not show a notable reproductive seasonality, in contrast to lemurs (Heldstab et al. 2021), could be another basis for the hypothesis that they are less prone to obesity in managed care.

In order to assess the degree to which the body masses of zoo-managed lemurs and colobine species deviate from those reported for their free-ranging conspecifics, we compared data collected from the global zoo community via the Species360 database with published literature reports. Based on the above considerations, our working hypothesis was that obesity is more prevalent in zoo-kept lemurs than in zoo-kept colobine monkeys.

## Methods

Data on the body mass of wild adult lemurs and colobine monkeys were taken from the scientific literature and online museum collections and are reported in detail in the supplementary materials (Tables S1.1 to S2.10). These data were used as benchmarks to visually compare and qualitatively analyse the data from the zoo populations.

As part of Species360 research data use agreement # 84212, we received data on body masses of lemurs and colobine species recorded in the Zoo Information Management System (ZIMS) and stored by Species360 in January 2024. Species-wise, our analysis does not extensively cover all species ever kept in zoos, but only those that had at least five individuals per sex (Tables 1 and 2). The entire dataset was anonymised, indicating only the body mass entered by a zoo, the date of record, a randomly assigned individual animal ID, sex, and the corresponding age of the animal, but not the identity of the reporting zoo (and hence, whether the dates refer to the seasons of the northern or southern hemisphere). Further details, such as health or reproductive status, were not included as part of the dataset. The dataset comprises both historical and current data, including deceased and living individuals. These raw data were provided with an indication of which data points were considered outliers by several automated correction procedures, as explained in Garand et al. (2024) and Meireles et al. (2025).

**Table 1:** Body mass (BM) for lemurs in the zoo data of the present study, compared to the mean body mass in natural habitats

Species	Sex	*n* (Zoo)	Mean Wild BM*	Mean (± SD) Zoo BM	Underweight	Healthy BM	Overweight	Obese	Very Obese
			kg		% of zoo individuals				
*Eulemur albifrons*	Female	40	1.94	2.20 ± 0.28	-	82.5	15.0	2.5	-
	Male	49	1.94	2.29 ± 0.44	-	63.3	28.6	4.1	4.1
*Eulemur collaris*	Female	42	2.15	2.47 ± 0.32	-	80.1	19.0	-	-
	Male	49	2.15	2.35 ± 0.20	-	95.9	4.1	-	-
*Eulemur coronatus*	Female	83	1.60	1.61 ± 0.18	2.4	98.8	-	-	-
	Male	111	1.60	1.65 ± 0.21	3.6	91.9	4.5	-	-
*Eulemur flavifrons*	Female	53	2.04	2.53 ± 0.31	-	49.1	47.2	3.8	-
	Male	62	1.87	2.41 ± 0.28	-	43.5	43.5	13.0	-
*Eulemur fulvus*	Female	66	1.92	2.48 ± 0.44	-	50.0	30.3	16.7	3.0
	Male	67	1.93	2.47 ± 0.36	-	52.2	34.3	11.9	1.5
*Eulemur macaco*	Female	126	1.86	2.61 ± 0.47	-	32.5	34.1	22.2	10.3
	Male	140	1.88	2.54 ± 0.41	-	34.2	43.6	15.7	6.4
*Eulemur mongoz*	Female	84	1.26	1.63 ± 0.24	-	45.2	41.2	8.3	4.8
	Male	93	1.38	1.57 ± 0.24	-	81.7	12.9	4.3	1.1
*Eulemur rubriventer*	Female	74	1.97	2.08 ± 0.29	-	93.2	4.1	2.7	-
	Male	72	1.99	2.03 ± 0.24	1.4	93.1	5.6	-	-
*Eulemur rufus*	Female	46	2.06	2.30 ± 0.35	-	84.8	8.7	6.5	-
	Male	52	1.97	2.30 ± 0.31	-	76.9	17.3	5.8	-
*Hapalemur alaotrensis*	Female	59	1.26	1.38 ± 0.27	1.7	78.0	18.6	1.7	1.7
	Male	74	1.23	1.40 ± 0.23	-	77.0	17.6	5.4	-
*Lemur catta*	Female	1624	2.12	2.76 ± 0.46	0.2	43.2	39.6	14.0	3.1
	Male	1858	2.14	2.87 ± 0.48	0.1	37.5	40.7	16.6	5.0
*Propithecus coquereli*	Female	56	3.78	4.17 ± 0.48	-	89.3	8.9	1.8	-
	Male	61	3.69	3.76 ± 0.36	-	96.7	3.3	-	-
*Varecia rubra*	Female	396	3.64	3.67 ± 0.55	3.3	89.6	6.8	0.3	-
	Male	515	3.30	3.45 ± 0.53	1.7	87.2	9.9	1.0	0.2
*Varecia variegata*	Female	527	3.52	3.77 ± 0.59	1.7	86.1	10.2	1.9	-
	Male	708	3.46	3.57 ± 0.51	2.5	90.7	7.6	0.3	-

*Weighted average, calculated from literature sources (see Tables S1.1–S1.14)

**Table 2:** Body mass (BM) for colobine monkeys in the zoo data of the present study, compared to the mean body mass in natural habitats

Species	Sex	*n* (Zoo)	Mean Wild BM*	Mean (± SD) Zoo BM	Underweight	Healthy BM	Overweight	Obese	Very Obese
			kg		% of zoo individuals				
*Colobus angolensis*	Female	68	7.87	10.17 ± 1.78	-	45.6	36.8	14.7	2.9
	Male	54	9.69	11.71 ± 1.75	-	59.3	33.3	9.3	-
*Colobus guereza*	Female	346	7.78	10.39 ± 1.98	-	41.0	37.0	15.3	6.6
	Male	301	9.64	12.65 ± 2.11	-	38.5	46.2	10.3	5.0
*Colobus polykomos*	Female	9	7.85	10.89 ± 1.61	-	33.3	33.3	33.2	-
	Male	6	9.49	12.75 ± 2.78	-	50.0	17.0	17.0	17.0
*Semnopithecus entellus*	Female	48	11.30	12.47 ± 3.02	10.4	64.6	17.0	6.3	2.1
	Male	17	14.23	17.50 ± 3.74	11.8	29.4	47.1	5.9	5.9
*Trachypithecus auratus*	Female	71	6.20	7.62 ± 1.44	-	62.0	28.2	5.6	4.2
	Male	39	7.10	8.35 ± 1.21	-	66.7	30.8	2.6	-
*Trachypithecus cristatus*	Female	57	5.80	6.80 ± 1.70	1.6	59.6	17.5	15.8	3.5
	Male	55	6.69	7.89 ± 1.55	-	65.5	21.8	10.9	1.8
*Trachypithecus francoisi*	Female	92	7.39	7.17 ± 1.01	2.2	92.4	5.4	-	-
	Male	105	7.80	7.97 ± 0.70	-	98.1	1.9	-	-
*Trachypithecus obscurus*	Female	60	6.46	6.76 ± 1.06	1.7	86.7	11.7	-	-
	Male	51	7.63	7.44 ± 1.09	-	94.1	5.9	-	-
*Nasalis larvatus*	Female	13	9.73	9.37 ± 1.08	7.7	92.3	-	-	-
	Male	5	20.60	17.63 ± 5.53	40.0	40.0	20.0	-	-
*Pygathrix nemaeus*	Female	27	8.44	8.05 ± 1.92	18.5	70.4	7.4	3.7	-
	Male	19	10.98	11.08 ± 2.69	-	84.2	5.3	10.5	-

*Weighted average, calculated from literature sources (see Tables S2.1–S2.10)

For each species, for females and males separately, a Gompertz growth model was fitted to the data. This model yields an asymptotic weight that can be interpreted as the growth plateau (Zullinger et al. 1984). Note that Gompertz models may not necessarily be the best models to fit growth data (reviewed in Veylit et al. 2021); here, we did not employ them to yield the most accurate data fit, but only to define the age at which animals typically reach adult size (= adult age; as defined and discussed by Smith and Jungers 1997). The adequacy of the models was checked by inspecting the resulting model as plotted against the raw data (Fig. S1). We defined the age from which on data would be included in the calculation of an adult average as 95% of this asymptotic mass. The resulting parameter estimates and ages used as the cut-off to define adulthood size are given in Table S3.

Based on the detected adult age threshold, the body mass was averaged per individual and then across the means of all individuals. After assessing the normality of sex-specific body mass data for males and females using the Shapiro–Wilk normality test, we tested for sexual dimorphism in the zoo population by an independent t-test when p (Shapiro–Wilk) > 0.05, and otherwise by a Mann–Whitney U test, using Python’s scipy package. Following a previous approach (Taylor et al. 2012; Mellor et al. 2020), we classified individuals into body mass categories based on how much the adult average body mass deviated from the weighted average body mass in the wild (determined by the values that we found in the literature). An individual is considered ‘underweight’ if it has 0.75 or less of the wild body mass. Between 0.75 and 1.25 times the wild body mass, an individual is considered to have a ‘healthy’ body mass. Between 1.25 and 1.5, it is considered ‘overweight’; between 1.5 and 1.75, ‘obese’; and above 1.75, ‘very obese’. We depicted this classification of body masses chronologically, from the first year with at least 10 individuals with data, calculating the proportion of the difference scores in the population to determine developments over time.

Additionally, two patterns were assessed visually by analysing the data for each individual animal separately: (i) regular, annual (i.e., seasonal) fluctuations in body mass, and (ii) a decrease in body mass towards later adult life. As a first step, only those individuals were selected for whom at least three measurements in both the first part of the expected lifetime and the second part of the expected lifetime were present. To assess seasonal fluctuations, pairs of measurements between one winter and the subsequent summer (or vice versa) were used. Finally, the data of these pre-selected individuals were plotted and all inspected individually, assessing subjectively if the detected pattern could be assessed based on the data distribution, and whether the pattern was visually evident (then it was counted) or not (then it was not). An example of such an individual plot is given in Fig. S2. Using this information, we determined the proportion of individuals that showed a seasonally fluctuating body mass and a decline in body mass with progressing age late in adult life.

## Results

Whereas the result plots for all species are provided in the supplement (Fig. S3-S5), we limit species plots in the results section to one example of a species with a large number of ‘overweight’, ‘obese’ or ‘very obese’ individuals (ring-tailed lemur, *L. catta*, and guereza colobus *C. guereza*) and one of a species with mainly individuals of ‘healthy’ body mass (red-bellied lemur *E. rubriventer*, and François’ langur *T. francoisi*) for both taxa.

### Lemurs

**Fig. 1:**
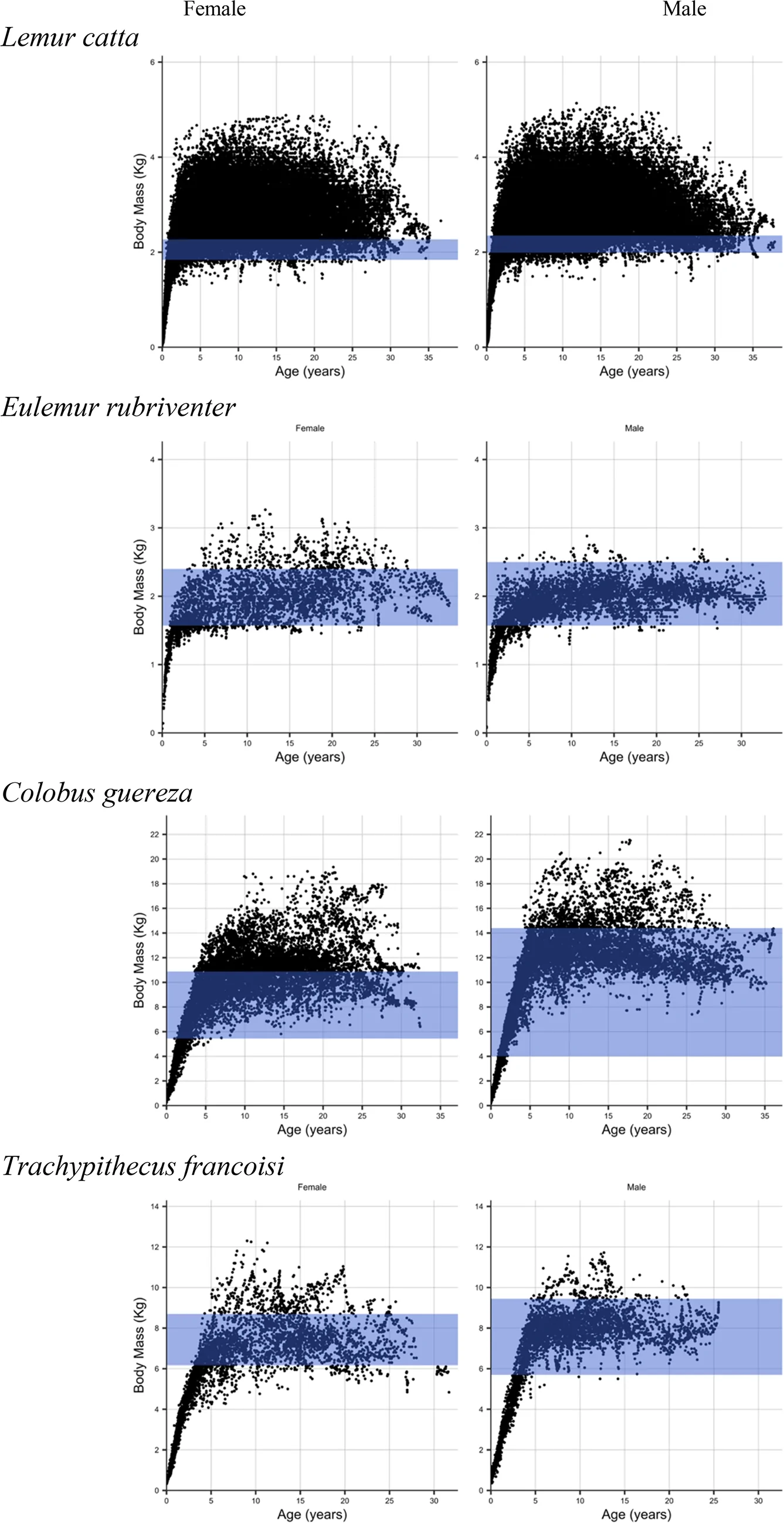
Body mass data for zoo-kept *Lemur catta*, *Eulemur rubriventer*, *Colobus guereza and Trachypithecus francoisi* (black dots) as compared to the literature data range of adult, free-ranging specimens (blue) (for sources, see Table S1.11, S1.8, S2.2 and S2.7). Note that this selection of individuals is based on data availability and not necessarily representative of the current global zoo populations

**Fig. 2:**
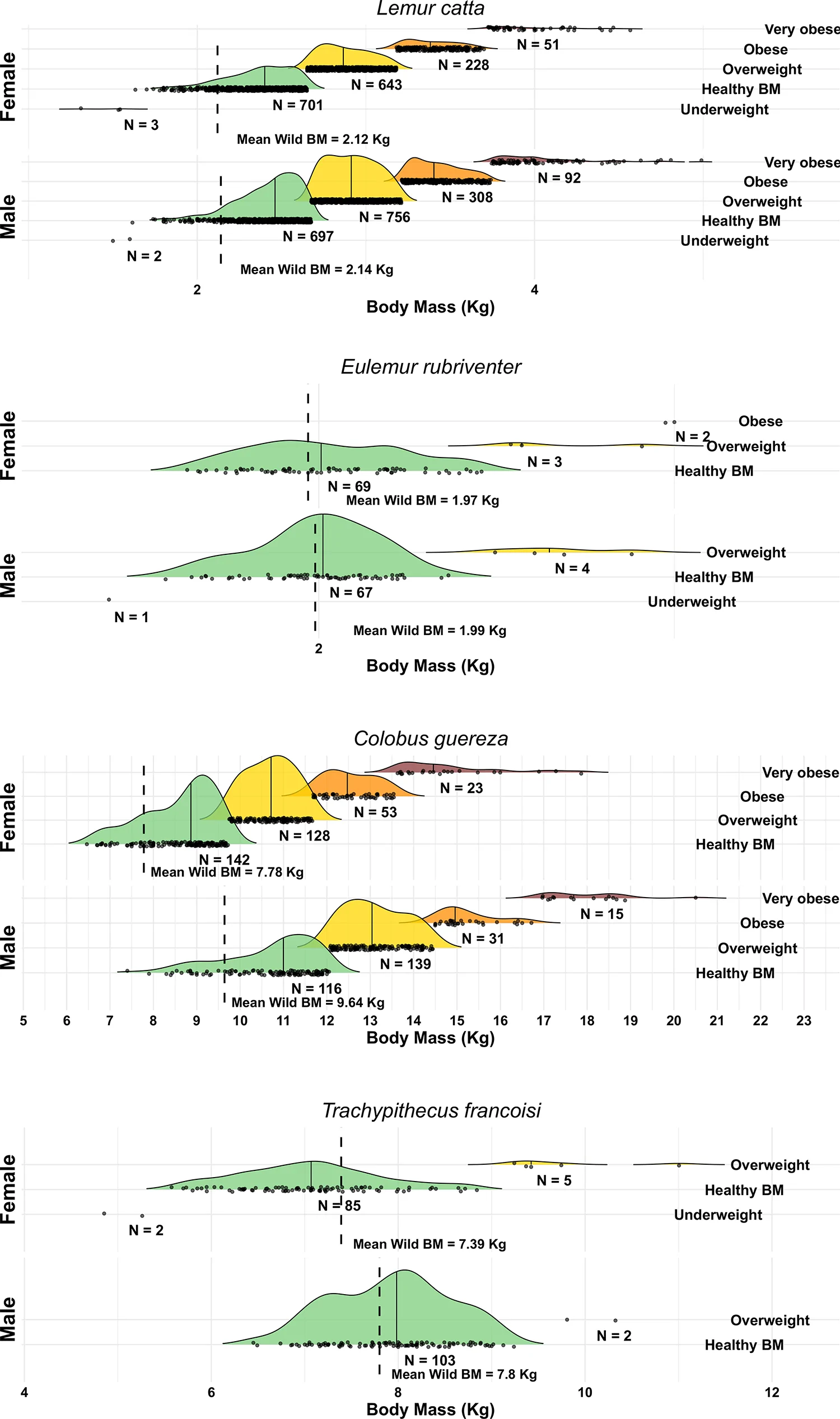
Distribution of densities of body mass scores across *Lemur catta*, *Eulemur rubriventer*, *Colobus guereza* and *Trachypithecus francoisi* individuals in zoos. Definitions in multiples of wild body mass: underweight < 0.75; healthy BM 0.75–1.25; overweight 1.25–1.5; obese 1.5–1.75

In general, the mean zoo body mass of the majority of lemur species was within the ‘healthy’ body mass range as compared to free-ranging specimens (Table 1)*.* Ring-tailed lemurs showed a remarkable difference in body mass between zoo and wild populations (Fig. 1, 2), with individuals in the ‘healthy’ BM range in the minority in our dataset (Table 1). The members of the genus *Eulemur* showed a more varied pattern in comparisons between zoo and wild animals (Fig. S3a, S4a). While white-headed (*E. albifrons),* collared brown* (E. collaris),* crowned *(E. coronatus)* (Fig. S3a, S4a), red-bellied* (E. rubiventer)* (Fig. 1, 2), and red *(E. rufus)* (Fig. S3a, S4a) lemurs were mostly within the ‘healthy’ BM range, the blue-eyed black (*E. flavifrons),* common brown (*E. fulvus),* black* (E. macaco),* and mongoose* (E. mongoz)* lemurs had a considerable number of animals that were well above the range of body masses found in wild populations (Table 1). Zoo individuals of Lac Alaotra gentle lemur (*H. alaotrensis)*, Coquerel’s sifaka (*P. coquereli)* and both ruffed lemur species (Genus *Varecia)* were again mostly within the ‘healthy’ BM range (Table 1, Fig. S3a, S4a). The percentage of adult animals that were ‘underweight’ compared to the free-range average was very low in lemur species, with a maximum in *E. coronatus* males at 4% (Table 1).

Sexual dimorphism in terms of body mass was evident for *E. collaris, E. flavifrons, E. mongoz, L. catta, P, coquereli, V. rubra* and *V. variegata*, but not in the other species (Table S4). Females were generally heavier than males in these cases (with male:female body mass ratios of 0.90–0.96), with the exception of *L. catta* where the males were heavier (1.04).

In females as well as males of all lemur species, seasonal body mass fluctuations prevailed in zoos, as did a general decline in body mass with old age (Table S4).

**Fig. 3:**
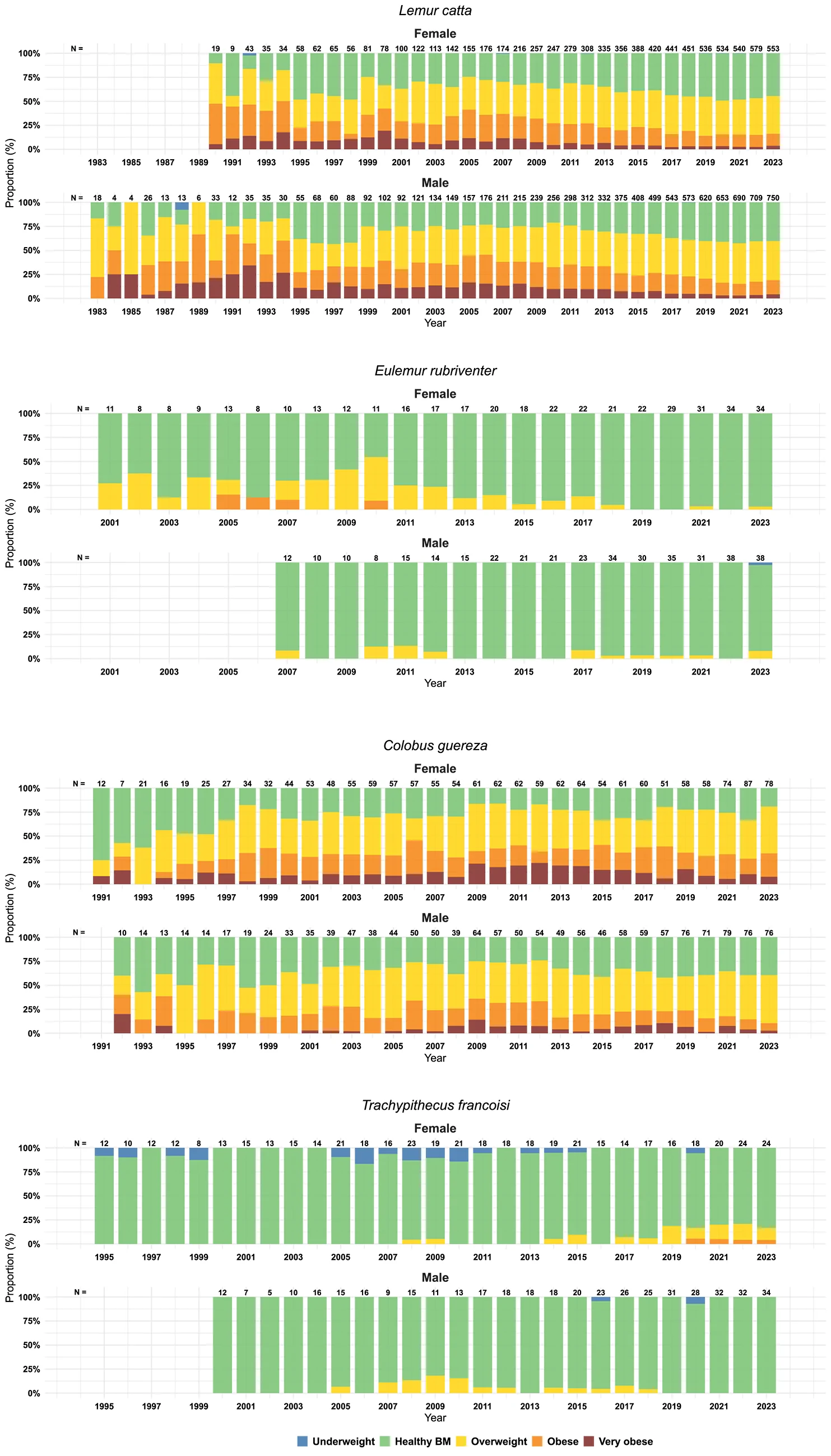
Historical trends (by year) of body mass category proportions in adult zoo-kept *Lemur catta*, *Eulemur rubriventer*, *Colobus guereza* and *Trachypithecus francoisi*. Data were used from the first year in which a minimum of 10 individuals were present in the dataset. Note that this selection of individuals is based on data availability (individuals weighed) and is not necessarily representative of the current global zoo populations. Definitions in multiples of wild body mass: underweight < 0.75; healthy BM 0.75–1.25; overweight 1.25–1.5; obese 1.5–1.75

**Fig. 4:**
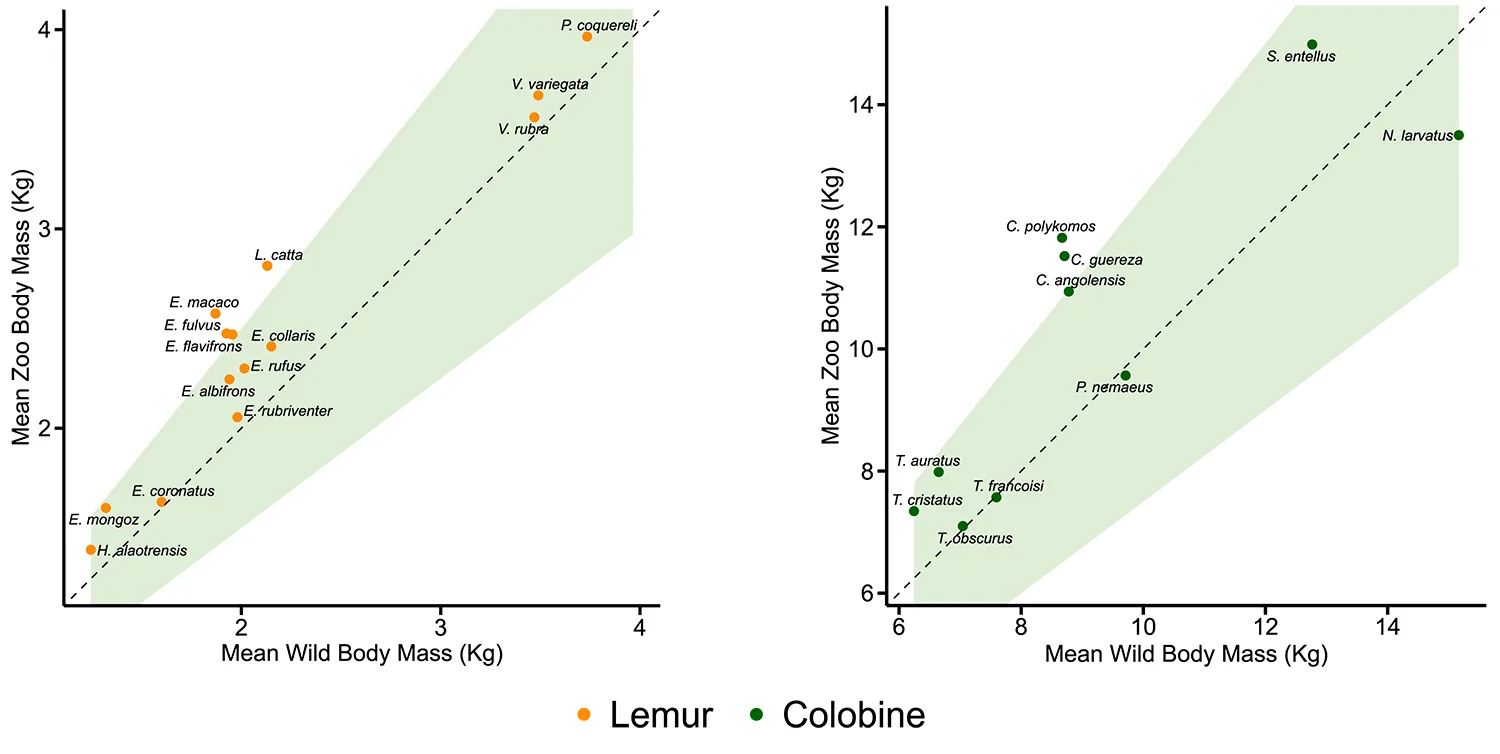
Association of the species’ mean body mass in the natural habitat compared to that recorded in zoos in the present study for lemurs (left) and colobines (right), with the green shaded area indicating the range of 25% around the y = x line. Note that the selection of zoo individuals is based on data availability (individuals weighed) and is not necessarily representative of the current global zoo populations

For *L. catta*, the historical development suggested some reduction of ‘overweight’ or ‘obese’ individuals over time, and a less distinct, similar pattern occurred in *E. rubriventer* (Fig. 3), *E. collaris* or *E. flavifrons* (Fig. S4a). By contrast, no improvement was apparent in *E. macaco* and *E. mongoz* (Fig. S4a). In summary, the mean zoo body mass of the majority of lemur species is within the ‘healthy’ body mass range as compared to free-ranging specimens, with *L. catta, E. macaco, E. flavifrons, E. fulvus* and female *E. mongoz* apparently most susceptible to being overweight (Fig. 4).

### Colobine monkeys

The majority of zoo individuals of most colobine monkeys was in the ‘healthy’ body mass range, i.e. did not show distinct deviations from body mass in zoos compared to the range of body masses from free-ranging populations (Fig. S3b, S4b), e.g. the François’ langur (*Trachipithecus francoisi)* (Fig. 1,2). However, the genus *Colobus*, e.g. the guereza colobus (Fig. 1,2), is a clear exception; the majority of the individuals of the three *Colobus* species were above the ‘healthy’ body mass range (Table 2). Individuals in the ‘underweight’ body mass range occurred at higher percentages (but note the low sample size) in the two species with quadripartite stomachs, *N. larvatus* (8–40%) and *P. nemaeus* (19%), as well as in the tripartite-stomach species Northern plains gray langur (*Semnopithecus entellus*) (10–12%) (Table 2).

Sexual dimorphism in terms of body mass with heavier males was evident in all colobine species and did not reach significance only in the King colobus (*Colubus polykomos)*, the species with the lowest sample size (Table S5). Male:female body mass ratios ranged from 1.10 in some *Trachypithecus* spp. to 1.88 in *N. larvatus*.

In general, colobine species did not show a seasonal body mass fluctuation in zoos with the exception of male *P. nemaeus*; as in lemurs, a decline in body mass with old age was prevalent in zoo colobines (Table S5).

In colobines, there were no evident historical developments with respect to obesity in zoos (Fig. 3, S5b).

In summary, the mean zoo body mass of the majority of colobine species is within the ‘healthy’ body mass range as compared to free-ranging specimens, with the guereza, king and Angolan colobus (*C. guereza, C. polykomos, C. angolensis)* and male *Semnopithecus entellus* apparently most susceptible to being overweight, and *N. larvatus* with a special propensity to be underweight (Fig. 4).

## Discussion

The present study continues a research tradition of comparing the body mass of zoo-kept primates to that of conspecifics in their natural habitats (Leigh 1994; Terranova and Coffman 1997; Goodchild and Schwitzer 2008; Taylor et al. 2012; Mellor et al. 2020; Pontzer 2023). In 5 out of 14 lemur species (36%), half of the individuals or more were above the ‘healthy’ body mass range in at least one sex, similar to the percentage of 5 out of 13 lemur species (38%) reported by Mellor et al. (2020). In the present study, this also applied to 4 out of 10 colobine species (40%). Thus, the results do not corroborate our hypothesis that obesity should occur distinctively less often in colobine species. In some congruence with our hypothesis, however, adults that were ‘underweight’ only occurred in some colobine species at higher percentages. Before these patterns are discussed in more detail, some limitations of the present study need to be mentioned.

### Study limitations and comparisons to previous studies

When using body mass as an indicator of nutritional status, caution is required. In humans, it is well-known that body mass must be related to body size (in the form of a body mass index) to yield a reliable indicator of body condition (WHO 2025). Most notably, the average human body mass has systematically increased with better living conditions, irrespective of obesity, because higher resource availability and better immunological status allow humans to use their full growth potential (Floud et al. 2011). Therefore, in humans, measuring body mass and body height at the same time is a standard procedure of every visit to a doctor. In zoo animals, the weighing procedure itself is already challenging for many institutions, requiring scales and some training or other logistical solutions that facilitate the positioning of a single animal on these scales. However, taking reliable length measurements that could be used to establish such a body mass index is most likely impossible in most zoo animals without anaesthesia and only practical in research settings (e.g., Sterck et al. 2019). Hence, body mass alone may not always be a true indicator of obesity in zoo animals but might also indicate more intense size growth as compared to natural conditions where resources may often be limited. This has, for example, been suggested as a possible explanation for the distinct body mass difference between free-ranging and zoo giant anteaters (*Myrmecophaga tridactyla*) (Garand et al. 2024), and a subsequent study suggested a combination of larger size and increased adipose deposits in zoo specimens (Pereira et al. 2025).

This problem has apparently received little attention in the literature on zoo primates; some authors do not mention it at all (e.g., Terranova and Coffman 1997), Mellor et al. (2020) acknowledge it, while Pontzer (2023) suggests that skeletal studies comparing primate individuals from natural habitats and zoos do not indicate a systematic difference, and claims that differences in body mass therefore directly translate into differences in nutritional status. This view is somewhat supported by a comparison of captive and free-ranging vervet monkeys (Turner et al. 2016). However, some evidence suggests larger skeletal structures in chimpanzees (*Pan troglodytes*) from human care as opposed to free-ranging specimens (Lewton 2017; Hanegraef and Spoor 2024). In primates, Leigh (1994) warns against uncritically interpreting high body masses in individuals that are known for lifelong skeletal growth, such as male orangutans (*Pongo* spp.). Without specific investigations that target representative skeletal samples of lemurs and colobines, we cannot resolve this dilemma, but we would expect this problem to affect lemurs and colobines alike. Nevertheless, we follow the common interpretation that the main reason for a discrepancy in body mass between free-ranging and zoo primates is a difference in the degree of obesity.

**Fig. 5:**
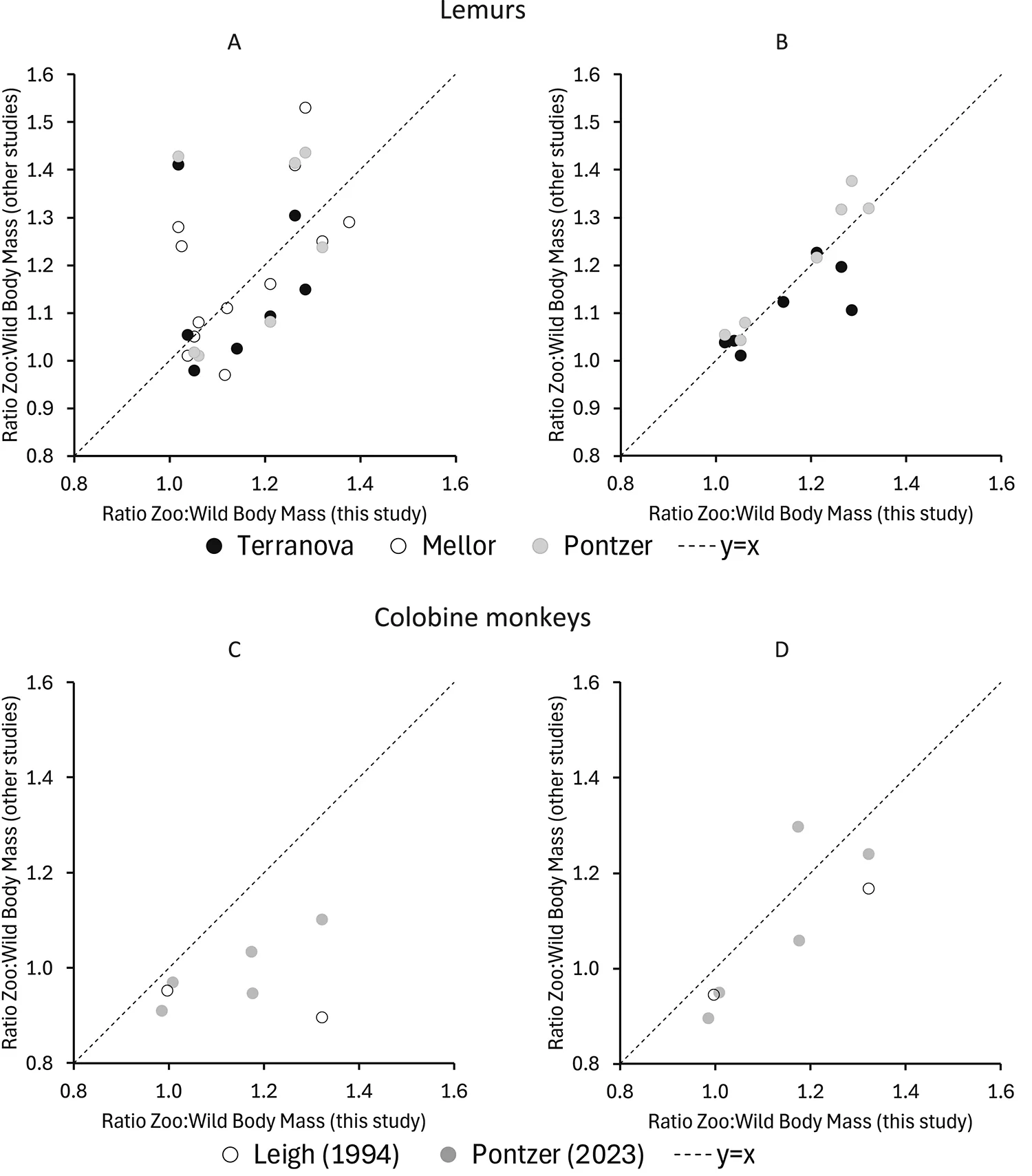
Comparison of the zoo:wild body mass ratio of the present study with that of previous studies using the other studies original data (left column, **A**, **C**) and the other studies original zoo data combined with the present study’s data for free-ranging specimens (right column, **B**, **D**). Because Mellor et al. (2020) do not give their body mass data but only the zoo:wild ratio, no correction can be applied to their data

**Fig. 6:**
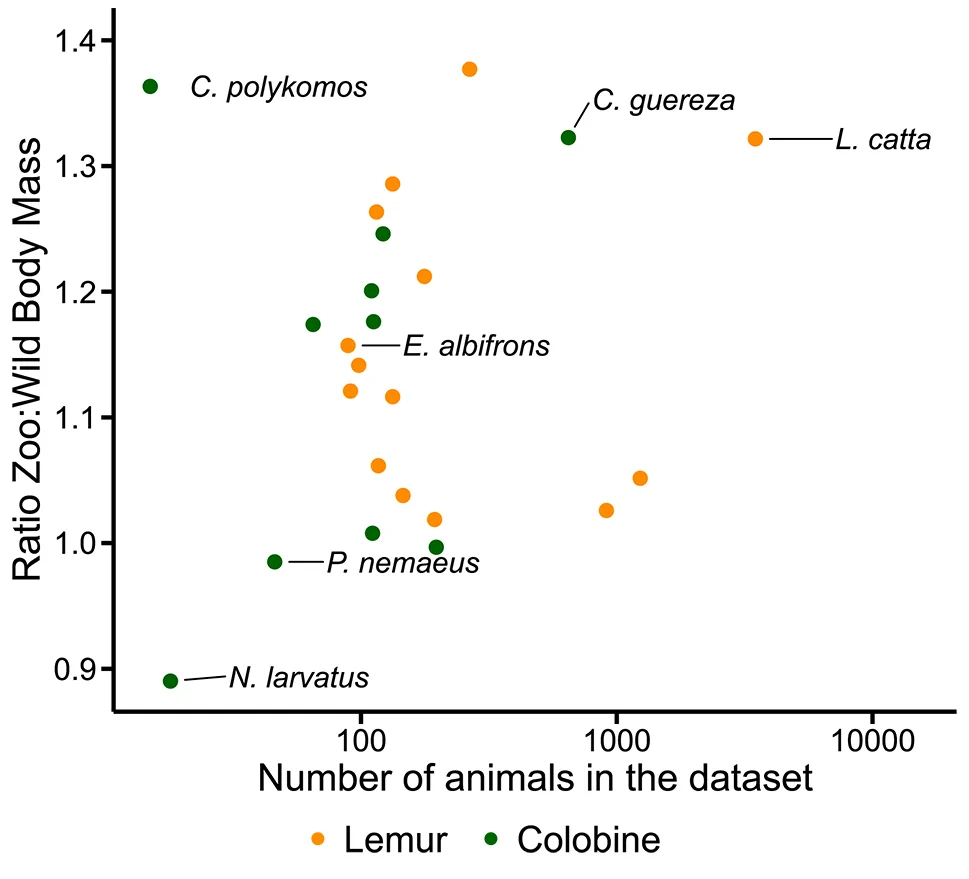
Absence of an effect of the dataset size on the propensity to obesity in zoos in the lemur and colobine species of the present study

When comparing our results to those of other studies (Tables 3–4, Fig. 5), some other aspects of primate body mass in zoos become evident. Differences in sample size (Table 3) may—as e.g. in the case of *E. fulvus*—or may not—as e.g. in the case of *L. catta* or *V. variegata*—influence the mean zoo body mass. Yet, in our dataset, there was no association between a species’ sample size and its perceived susceptibility to obesity in zoos (Fig. 6). Yet, we acknowledge that some species have a remarkably small sample size, which could be derived from a single zoo institution, hence reflecting that institution’s practices instead of a representation of the species as a whole. Additionally, differences in the wild body mass distilled from literature sources can have an important effect on how a species is considered. For example, Terranova and Coffman (1997) and Pontzer (2023) assume a natural body mass of 1.18 kg for *E. coronatus*, leading to a high zoo:wild ratio in this species, whereas we set this as 1.60 kg, based on the literature sources we surveyed (Table S1.3). Thus, when applying our wild body mass to calculate zoo:wild ratios for the data in the literature, the results between the studies are generally in better alignment (Fig. 5), indicating how easily affected species-specific interpretations are by these baseline decisions. The intricacies of realistically homing in on the true natural body mass of primate species have been discussed in detail by Smith and Jungers (1997). Given these uncertainties, species-specific characteristics that are systematically linked to obesity susceptibility in human care may be difficult to detect reliably.

**Table 3:** Comparison of the mean zoo body mass (kg) and the ratio of mean zoo:wild body mass in lemurs between the present and previous studies

Source	This study			Schwitzer and Kaumanns (2001)			Terranova and Coffman (1997)			Mellor et al. (2020)*		Pontzer (2023)		
Species	*n*	Zoo mean	Zoo:Wild	*n*	Zoo mean	Zoo:Wild	*n*	Zoo mean	Zoo:Wild	*n*	Zoo:Wild	*n*	Zoo mean	Zoo:Wild
*Eulemur albifrons*	89	2.25	1.16											
*Eulemur collaris*	91	2.41	1.12							17	1.11			
*Eulemur coronatus*	194	1.63	1.02				30	1.66	1.41	21	1.28	37	1.69	1.43
*Eulemur flavifrons*	115	2.47	1.26				21	2.34	1.30	13	1.41	48	2.58	1.41
*Eulemur fulvus*	133	2.48	1.29				17	2.13	1.15	11	1.53	24	2.65	1.44
*Eulemur macaco*	266	2.58	1.38							14	1.29			
*Eulemur mongoz*	177	1.60	1.21				67	1.62	1.09	29	1.16	56	1.61	1.08
*Eulemur rubriventer*	146	2.06	1.04				15	2.06	1.05	14	1.01			
*Eulemur rufus*	98	2.30	1.14				41	2.26	1.02					
*Hapalemur alaotrensis*	133	1.39	1.12							12	0.97			
*Lemur catta*	3482	2.82	1.32							351	1.25	144	2.81	1.24
*Propithecus coquereli*	117	3.97	1.06							17	1.08	71	4.03	1.01
*Varecia rubra*	911	3.56	1.03							75	1.24			
*Varecia variegata*	1235	3.67	1.05	43	4.38	1.31	53	3.52	0.98	89	1.05	96	3.64	1.02

*Mellor et al. (2020) do not indicate the mean zoo BM recorded in their study but only the Zoo:Wild ratio

**Table 4:** Comparison of the mean zoo body mass (kg) and the ratio of mean zoo:wild body mass in colobine monkeys between the present and previous studies

Source	This study			Leigh (1994)*		Pontzer (2023)		
Species	*n*	Zoo mean	Zoo:Wild	Zoo mean	Zoo:Wild	*n*	Zoo mean	Zoo:Wild
*Colobus angolensis*	122	10.94	1.25					
*Colobus guereza*	647	11.52	1.32	10.8	1.10	7	10.17	0.90
*Colobus polykomos*	15	11.82	1.36					
*Semnopithecus entellus*	65	14.99	1.17	16.57	1.03			
*Trachypithecus auratus*	110	7.99	1.20					
*Trachypithecus cristatus*	112	7.35	1.18	6.61	0.95			
*Trachypithecus francoisi*	197	7.57	1.00			34	7.17	0.95
*Trachypithecus obscurus*	111	7.10	1.01	6.68	0.97			
*Nasalis larvatus*	18	13.50	0.89	14.1	0.69**			
*Pygathrix nemaeus*	46	9.57	0.99	8.69	0.91			

*Leigh (1994) does not indicate the sample size

**For *N. larvatus*, Leigh (1994) only has data for males

Finally, it must be noted that we did not have information on the reproductive status of females, and seasonal fluctuations in female body mass may well reflect seasonal accretion of foetus, placenta and associated tissues. However, given that males similarly showed seasonal fluctuations, we suggest these findings also indicate a generally seasonal physiology.

### No associations between species characteristics and putative obesity in zoos

**Fig. 7:**
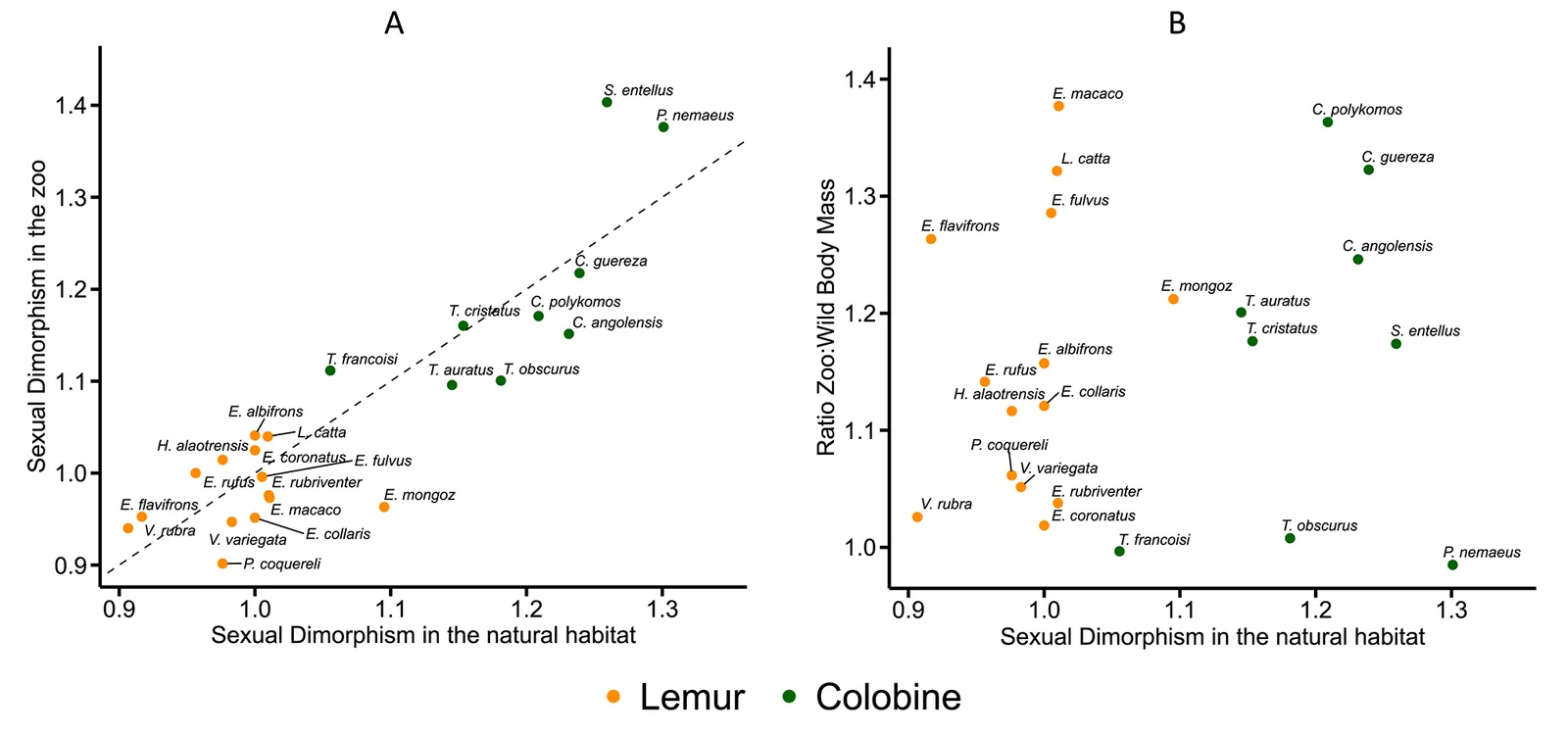
Comparison of the sexual dimorphism in the natural habitat (from Heldstab et al. 2021) with **A** the sexual dimorphism in zoos and **B**the propensity for obesity in lemur and colobine species in zoos

**Fig. 8:**
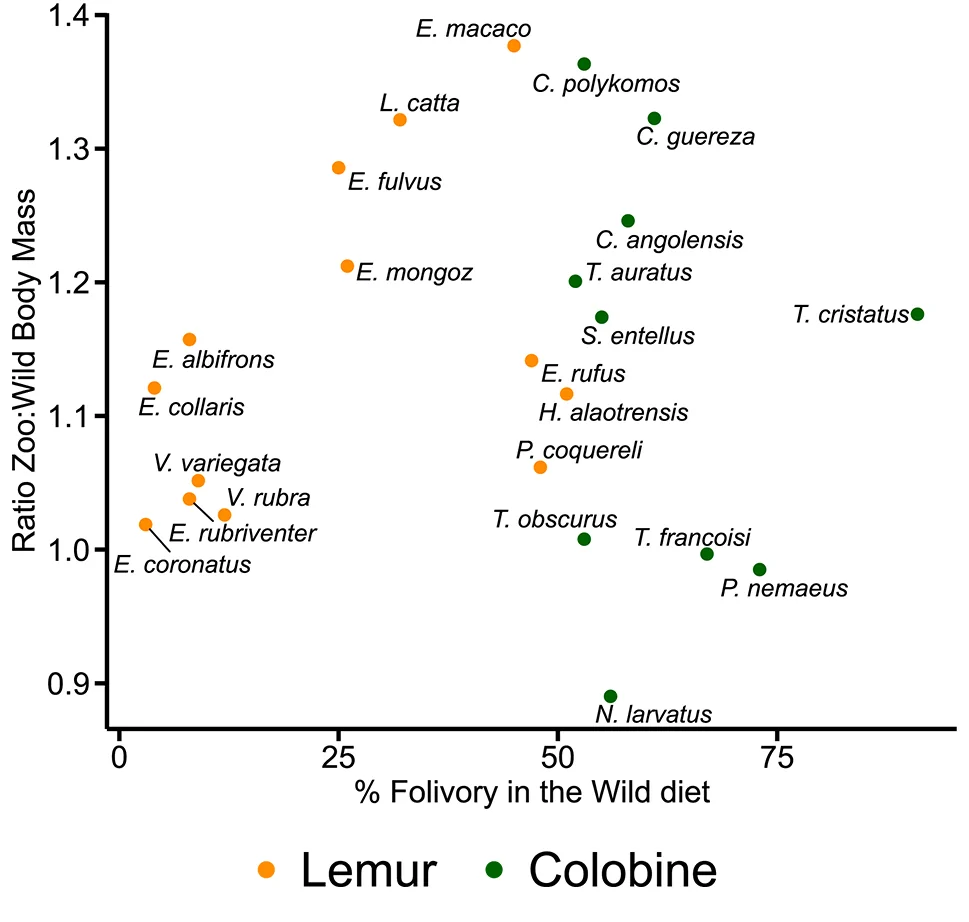
Comparison of the degree of folivory in the natural habitat (from Heldstab et al. 2021) with the the propensity for obesity in lemur and colobine species in zoos

Following Mellor et al. (2020), we assessed several putative associations with the magnitude of the zoo:wild body mass ratio as a proxy for the degree of overweight in zoos. Generally, the patterns of sexual body mass dimorphism typical for a certain species were preserved in zoos (Fig. 7a), and there was no association between the degree of sexual dimorphism in natural habitats with the zoo overweight (Fig. 7b). The degree of folivory in the natural habitat ascribed to species showed no association with zoo overweight, suggesting that a putative adaptation to fruit or leaves as the major staple natural diet items bears little relevance for zoo obesity (Fig. 8).

**Fig. 9:**
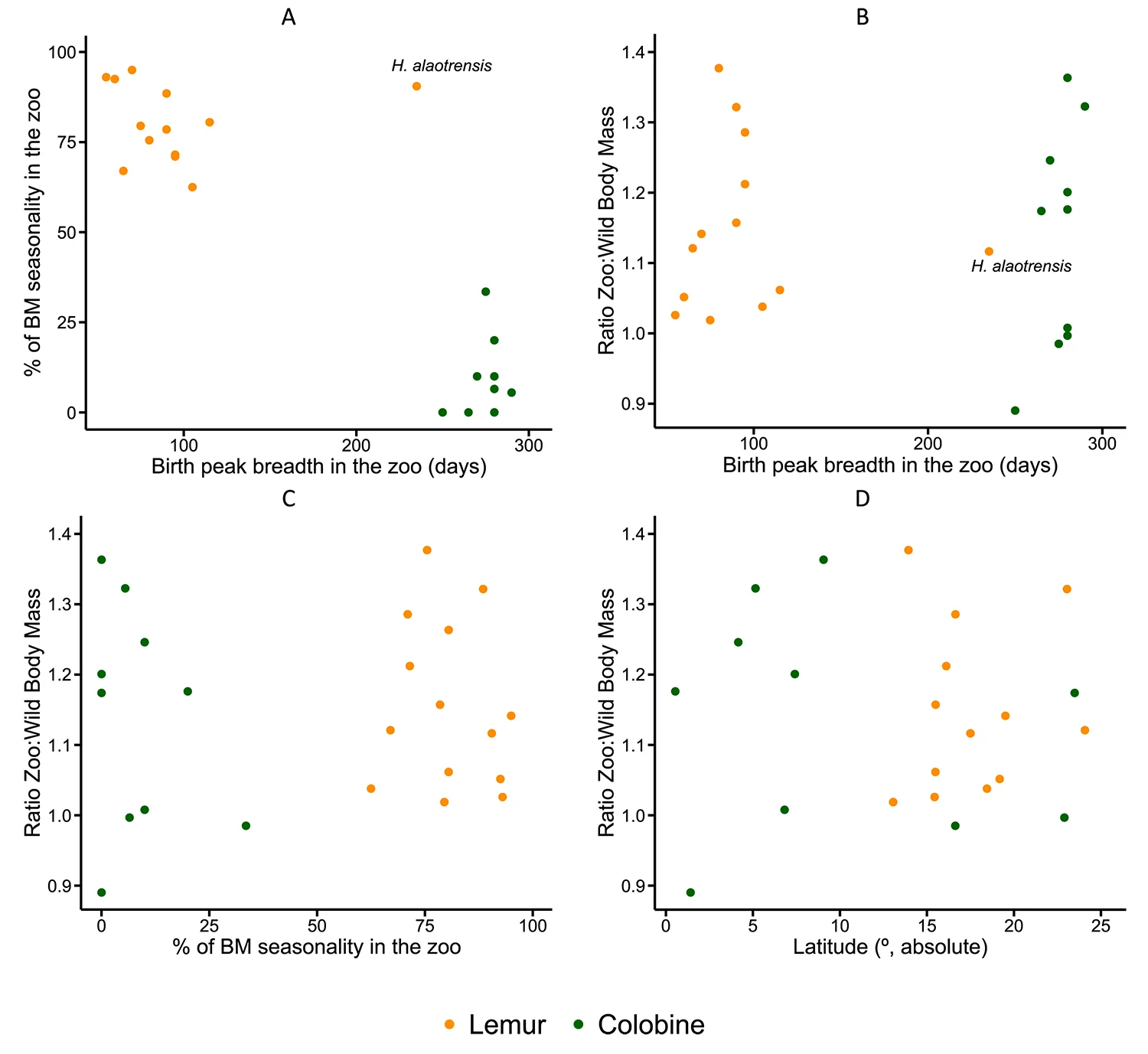
Measures relating to a seasonal physiology in lemurs and colobines. **A** Comparison of a measure for seasonal reproduction ((Birth Peak Breadth BPB80 in zoos from Heldstab et al. 2021) with the percentage of individuals with a seasonal body mass fluctuation in the present study (Tables S4, S5); and comparisons of the propensity for zoo obesity with **B** the BPB80, **C** the percentage of individuals with a seasonal body mass fluctuation and **D** the absolute latitude of a species’ natural habitat (from Heldstab et al. 2021)

A particular striking observation in the present study was the distinct difference in that lemurs in zoos showed seasonally varying body mass, whereas colobine monkeys did not (Table S4, S5). When plotting the average percentage of individuals that showed a seasonally fluctuating body mass against a species-specific measure for reproductive seasonality (the birth peak breadth, BPB80, Heldstab et al. 2021), there is a close association between the two, suggesting that species with a seasonal reproduction (lemurs) also show a seasonally fluctuating body mass in zoos (Fig. 9A). Actually, the lemurs’ general seasonal physiology had been one of the reasons why we had suggested that they should be more prone to obesity in zoos (see Introduction). However, the susceptibility to zoo obesity was not associated with reproductive seasonality (Fig. 9B), body mass seasonality (Fig. 9C), nor the absolute average latitude of the species’ natural habitat range (Fig. 9D), which is highly correlated to seasonal physiology (Heldstab et al. 2021). We consider the physiological capacity of seasonal fat accretion a potential reason for the susceptibility of a species to obesity under captive conditions, but note that this mechanism is not sufficient to explain differences between lemur species, or why colobine species from less seasonal habitats can also become obese in zoos. We do not follow Mellor et al. (2020) in the speculation that between-year resource variability in the natural habitat could represent an evolutionary selective driver for fat accretion beyond that of seasonal resource variability, making species originating from such habitats more susceptible to zoo obesity than lemurs from (seasonal) habitats with less intra-annual variability. Considering the relatively low body mass of lemurs, we consider it unlikely that adipose tissue can contribute to buffer resource variation over periods of a year (Lindstedt and Boyce 1985).

In conclusion, the data of the present study did not yield evidence for an explanation of zoo obesity patterns by biological species characteristics. We refrain from *ad hoc* speculations why those species identified here as particularly prone to higher body masses in zoos than in the wild show that characteristic; detailed analyses of biological as well as husbandry factors applying particularly to these as opposed to other species would be welcome.

### Primate foreguts and obesity

In contrast to our hypothesis, the presence of a foregut was also not associated with a generally low probability of body masses in zoos above those reported in the wild. The hypothesis followed the logic that low-fibre diets high in easily digestible carbohydrates, as are often used in zoo primate nutrition (Clauss et al. 2025), should lead to the direct digestion and absorption of easily digestible components in the small intestine of hindgut fermenters, leading to obesity. In foregut fermenters, by contrast, the easily digestible carbohydrates reach the site of microbial fermentation before they could be digested in the small intestine, and hence might lead to excessive fermentation similar to acidosis in domestic ruminants (Nijboer and Clauss 2006; Clauss and Dierenfeld 2008), including consequences like chronically low intakes or ill thrift. The findings in *Colobus* spp., however, do not corroborate this theory. This most likely reflects situations where the provision of easily fermentable carbohydrates in zoos is of a degree that allows fat accretion, while often not being so excessive as to cause acute malfermentation.

The only anatomy-based assumption that is not outright rejected by our data refers to the number of compartments in the colobine stomach. Two types of colobine forestomachs have been described (Matsuda et al. 2019, 2022b): the genera *Colobus*, *Semnopithecus* and *Trachypithecus* have so-called ‘tripartite’ stomachs with three chambers, whereas *Nasalis* and *Pygathrix* have ‘quadripartite’ stomachs with an additional chamber that represents a dead-end expansion. Because this structure might allow higher instantaneous food intakes at no increased digesta flow, it has been suggested that colobines with quadripartite stomachs might be particularly susceptible to the effects of too-easily digestible diets, in line with observations across zoos that *Nasalis* and *Pygathrix* species have a history of particularly poor husbandry success in zoos (Matsuda and Clauss 2022). In our data, *N. larvatus* and *P. nemaeus* had a comparatively high proportion of underweight specimens (Table 2), similar to the observations of Hoshino et al. (2023) in *N. larvatus* in a Japanese zoo, which would match the theory. However, the tripartite species *Semnopithecus entellus* also had 10% of underweight specimens (Table 2), indicating that this morphology-based explanation cannot explain all observations. Thus, further research on the susceptibility of colobine monkeys to low body masses is warranted. Possibly, a propensity for renal disease and urolithiasis (Baker et al. 2022; Hoshino et al. 2023), the causes of which are not fully resolved yet, might play a role in chronic underweight in affected specimens.

### Historical developments and future dietary solutions

The data available for the present study did not give evidence for distinct changes in the body mass status of lemurs or colobines over time. This might have been expected for colobines. By contrast, the long history of publications that warn about lemur obesity (Schaaf and Stuart 1983; Pereira and Pond 1995; Terranova and Coffman 1997; Schwitzer and Kaumanns 2001, 2009; Goodchild and Schwitzer 2008; Taylor et al. 2012; Mellor et al. 2020; Pontzer 2023) could have been expected to have led to a higher awareness of the issue and more distinct reductions average body mass in the most recent years. We interpret the tentative increase in the proportion of ‘healthy’ individuals and, in particular, the reduction in ‘obese’ and ‘very obese’ individuals in *L. catta* as a positive development in this respect that should be reinforced.

The diets of zoo primates are generally characterized by comparatively low fibre levels (Nijboer and Dierenfeld 1996; Feistner and Mutschler 1999; Schwitzer et al. 2006a; Polowinsky and Schwitzer 2009; Cabana et al. 2018a, b; Matsuda et al. 2018). The most promising approach to a general reduction in primate obesity in zoos is most likely an increase in diet fibre levels to similar ones as observed in natural habitats, a measure that will automatically reduce the level of easily digestible—and highly palatable—carbohydrates in zoo diets (reviewed in Clauss et al. 2025). Simply restricting the amounts of food is typically not a suitable option in primates that are mostly kept and fed in groups, as dominant individuals might monopolize food, leading to undernutrition of subordinate individuals. By contrast, providing high-fibre food items such as browse, green leafy vegetables and pelleted or extruded compound feeds truly high in fibre should lead to less food monopolisation, especially if only one kind of item is offered per feeding rather than a mixture, and will allow animals to meet their energy and nutrient requirements without an incentive for overconsumption (Clauss et al. 2025). A very important step in this process is to realize that the reasons for proactively recommending lower dietary fibre levels than reported in natural diets (as done in the NRC 2003) no longer reflect realities of feed production capabilities, and likely do not align with biologically-founded nutritional targets of zoos (Clauss et al. 2025).

## Supplementary Information

Below is the link to the electronic supplementary material.

Supplementary Material 1

## Data Availability

The data that supports the findings of this study are available in the supplementary material of this article.
