# Supplementary material for: No systematic difference in obesity susceptibility between two groups of zoo primates with distinct digestive physiologies: hindgut-fermenting lemurs and foregut-fermenting colobines
Source: Primates. 2026 Feb 21;67(3):313–29. doi: 10.1007/s10329-026-01245-4 (PMC13668938; doi:10.1007/s10329-026-01245-4)
Supplement: Supplementary file 1 [file 10329_2026_1245_MOESM1_ESM.docx]

***Supplementary material***

**No systematic difference in obesity susceptibility between two groups of zoo primates with distinct digestive physiologies: hindgut-fermenting lemurs and foregut-fermenting colobines**

Christiane Krauss, Elisa Garand, Max Hahn-Klimroth, Dennis W. H. Müller, Paul W. Dierkes, Ikki Matsuda, Marcus Clauss, João Pedro Meireles

**Table S1.1** Body mass records (in kg) for adult, free-ranging white-headed lemur (*Eulemur albifrons*) in natural habitats

| **Source** | **n** | **minimum** | **average** | **maximum** |
| --- | --- | --- | --- | --- |
|  |  | *females* | | |
| Gordon et al. (2016) |  |  |  |  |
| Population 1 | 6 |  |  | 2.30 |
| Population 2 | 5 |  |  | 1.75 |
|  |  |  |  |  |
|  |  | *males* | | |
| Gordon et al. (2016) |  |  |  |  |
| Population 1 | 5 |  |  | 2.35 |
| Population 2 | 4 |  |  | 1.70 |
|  |  |  |  |  |
|  |  | *unknown sex* | | |
| Junge et al. (2008) | 10 |  | 2.05 |  |
|  | 7 |  | 2.31 |  |
|  | 7 |  | 1.57 |  |
|  | 10 |  | 1.68 |  |
|  |  |  |  |  |

**Table S1.2** Body mass records (in kg) for adult, free-ranging collared brown lemur (*Eulemur collaris*) in natural habitats

| **Source** | **n** | **minimum** | **average** | **maximum** |
| --- | --- | --- | --- | --- |
|  |  | *females* | | |
| Gordon et al. (2016) |  |  |  |  |
| Population 1 | 4 |  |  | 2.10 |
| Population 2 | 4 |  |  | 2.60 |
| Population 3 | 4 |  |  | 2.40 |
| Population 4 | 16 |  |  | 2.80 |
|  |  |  |  |  |
|  |  | *males* | | |
| Gordon et al. (2016) |  |  |  |  |
| Population 1 | 6 |  |  | 2.50 |
| Population 2 | 6 |  |  | 2.50 |
| Population 3 | 6 |  |  | 2.50 |
| Population 4 | 11 |  |  | 2.60 |
|  |  |  |  |  |
|  |  | *unknown sex* | | |
| Donati et al. (2011) | 11 |  | 2.15 |  |
|  |  |  |  |  |

**Table S1.3** Body mass records (in kg) for adult, free-ranging crowned lemur (*Eulemur coronatus*) in natural habitats

| **Source** | **n** | **minimum** | **average** | **maximum** |
| --- | --- | --- | --- | --- |
|  |  | *females* | | |
| Gordon et al. (2016) |  |  |  |  |
| Population 1 | 6 |  |  | 1.60 |
| Population 2 | 19 |  |  | 1.65 |
| Population 3 | 15 |  |  | 1.60 |
| Smith and Jungers (1997) | 14 |  | 1.62 |  |
|  |  |  |  |  |
|  |  | *males* | | |
| Gordon et al. (2016) |  |  |  |  |
| Population 1 | 4 |  |  | 1.10 |
| Population 2 | 13 |  |  | 1.55 |
| Population 3 | 10 |  |  | 1.60 |
| Smith and Jungers (1997) | 16 |  | 1.62 |  |
|  |  |  |  |  |
|  |  | *unknown sex* | | |
| Steffens et al. (2022) | 7 |  | 1.36 |  |
| Zaonarivelo et al. (2007) | 2 |  | 1.20 |  |
|  |  |  |  |  |

**Table S1.4** Body mass records (in kg) for adult, free-ranging blue-eyed black lemur (*Eulemur flavifrons*) in natural habitats

| **Source** | **n** | **minimum** | **average** | **maximum** |
| --- | --- | --- | --- | --- |
|  |  | *females* | | |
| Randriatahina and Roeder (2012) | 9 |  | 2.04 |  |
|  |  |  |  |  |
|  |  | *males* | | |
| Randriatahina and Roeder (2012) | 12 |  | 1.87 |  |
|  |  |  |  |  |
|  |  | *unknown sex* | | |
| Terranova and Coffman (1997) | 7 | 1.60 | 1.79 | 2.18 |
|  |  |  |  |  |

**Table S1.5** Body mass records (in kg) for adult, free-ranging common brown lemur (*Eulemur fulvus*) in natural habitats

| **Source** | **n** | **minimum** | **average** | **maximum** |
| --- | --- | --- | --- | --- |
|  |  | *females* | | |
| Gordon et al. (2016) | 15 |  |  | 2.45 |
| Simmen et al. (2010) | 6 | 1.58 | 1.786 | 2.16 |
| Smith and Jungers (1997) | 13 |  | 2.25 |  |
| Bauchot and Stephan (1966) | 2 |  | 1.69 |  |
| Andriantompohavana et al. (2006) | 7 |  | 1.5 |  |
|  |  |  |  |  |
|  |  | *males* | | |
| Gordon et al. (2016) | 11 |  |  | 3.00 |
| Simmen et al. (2010) | 9 | 1.58 | 1.90 | 2.17 |
| Sato (2013) | 1 |  | 1.92 |  |
| Smith and Jungers (1997) | 20 |  | 2.18 |  |
| Bauchot and Stephan (1966) | 6 |  | 1.30 |  |
| Andriantompohavana et al. (2006) | 3 |  | 1.60 |  |
|  |  |  |  |  |
|  |  | *unknown sex* | | |
| Rasmussen (2005) | 7 |  | 1.77 |  |
| Bradley et al. (1997) | 12 |  | 2.10 |  |
|  |  |  |  |  |

**Table S1.6** Body mass records (in kg) for adult, free-ranging black lemur (*Eulemur macaco*) in natural habitats

| **Source** | **n** | **minimum** | **average** | **maximum** |
| --- | --- | --- | --- | --- |
|  |  | *females* | | |
| Bayart and Simmen (2005) | 9 |  | 1.99 |  |
| Junge and Louis (2007) | 11 | 0.80 | 1.75 | 2.35 |
| Gordon et al. (2016) | 14 |  |  | 2.20 |
|  |  |  |  |  |
|  |  | *males* | | |
| Bayart and Simmen (2005) | 9 |  | 2.03 |  |
| Junge and Louis (2007) | 14 | 1.10 | 1.78 | 2.20 |
| Gordon et al. (2016) | 11 |  |  | 2.35 |

**Table S1.7** Body mass records (in kg) for adult, free-ranging mongoose lemur (*Eulemur mongoz*) in natural habitats

| **Source** | **n** | **minimum** | **average** | **maximum** |
| --- | --- | --- | --- | --- |
|  |  | *females* | | |
| Smith and Jungers (1997) | 4 |  | 1.41 |  |
| Gordon et al. (2016) | 11 |  |  | 1.20 |
|  |  |  |  |  |
|  |  | *males* | | |
| Smith and Jungers (1997) | 4 |  | 1.56 |  |
| Gordon et al. (2016) | 9 |  |  | 1.30 |
|  |  |  |  |  |
|  |  | *unknown sex* | | |
| Terranova and Coffman (1997) | 8 | 1.22 | 1.48 | 1.68 |
| Rasmussen (2005) | 2 |  | 0.99 |  |
|  |  |  |  |  |

**Table S1.8** Body mass records (in kg) for adult, free-ranging red-bellied lemur (*Eulemur rubriventer*) in natural habitats

| **Source** | **n** | **minimum** | **average** | **maximum** |
| --- | --- | --- | --- | --- |
|  |  | *females* | | |
| Gordon et al. (2016) |  |  |  |  |
| Population 1 | 24 |  |  | 2.40 |
| Population 2 | 7 |  |  | 2.25 |
| Population 3 | 5 |  |  | 2.19 |
| Population 4 | 7 |  |  | 2.20 |
| Glander et al. (1992) | 14 |  | 1.97 |  |
|  |  |  |  |  |
|  |  | *males* | | |
| Gordon et al. (2016) |  |  |  |  |
| Population 1 | 26 |  |  | 2.50 |
| Population 2 | 8 |  |  | 2.35 |
| Population 3 | 4 |  |  | 2.30 |
| Population 4 | 7 |  |  | 2.34 |
| Glander et al. (1992) | 11 |  | 1.99 |  |
|  |  |  |  |  |
|  |  | *unknown sex* | | |
| E.Balko unpublished Data, DUPC capture report, cited in Terranova and Coffman (1997) | 29 | 1.57 | 1.95 | 2.4 |
|  |  |  |  |  |

**Table S1.9** Body mass records (in kg) for adult, free-ranging red lemur (*Eulemur rufus*) in natural habitats

| **Source** | **n** | **minimum** | **average** | **maximum** |
| --- | --- | --- | --- | --- |
|  |  | *females* | | |
| Gerson (1999) western population | - |  | 1.84 |  |
| Johnson et al. (2005) | 15 |  | 2.29 |  |
| Junge and Louis (2005) | 7 | 1.0 | 1.26 | 1.60 |
| Gordon et al. (2016) | 13 |  |  | 1.60 |
| Glander et al. (1992) | 14 |  | 2.24 |  |
|  |  |  |  |  |
|  |  | *males* | | |
| Gerson (1999) western population | - |  | 1.79 |  |
| Johnson et al. (2005) | 23 |  | 2.13 |  |
| Junge and Louis (2005) | 13 | 1.20 | 1.41 | 1.60 |
| Gordon et al. (2016) | 7 |  |  | 1.60 |
| Glander et al. (1992) | 20 |  | 2.17 |  |
|  |  |  |  |  |
|  |  | *unknown sex* | | |
| Gerson (1999) |  |  |  |  |
| Eastern Population |  |  | 2.21 |  |

**Table S1.10** Body mass records (in kg) for adult, free-ranging bamboo lemur (*Hapalemur alaotrensis*) in natural habitats

| **Source** | **n** | **minimum** | **average** | **maximum** |
| --- | --- | --- | --- | --- |
|  |  | *females* | | |
| Mutschler et al. (2000) | 27 |  | 1.25 |  |
| Rabarivola et al. (2007) | 1 |  | 1.55 |  |
|  |  |  |  |  |
|  |  | *males* | | |
| Mutschler et al. (2000) | 31 |  | 1.22 |  |
| Rabarivola et al. (2007) | 2 |  | 1.35 |  |
|  |  |  |  |  |
|  |  | *unknown sex* | | |
| Mutschler et al. (2000) | 31 | 0.90 | 1.23 | 1.60 |
|  |  |  |  |  |

**Table S1.11** Body mass records (in kg) for adult, free-ranging ring-tailed lemur (*Lemur catta*) in natural habitats

| **Source** | **n** | **minimum** | **average** | **maximum** |
| --- | --- | --- | --- | --- |
|  |  | *females* | | |
| Ichino et al. (2013) |  |  |  |  |
| Year 1999 | 30 |  | 2.27 |  |
| Year 2006 | 46 |  | 1.98 |  |
| Year 2011 | 20 |  | 1.99 |  |
| Sauther et al. (2006) | n.g. |  | 2.25 |  |
| Sussman (1991) | 24 |  | 2.20 |  |
| Simmen et al. (2010) |  |  |  |  |
| Year 2006 | 4 |  | 2.18 |  |
| Year 2005 | 1 |  | 1.84 |  |
| Rainwater et al. (2009) | 25 |  | 2.21 |  |
|  |  |  |  |  |
|  |  | *males* | | |
| Ichino et al. (2013) |  |  |  |  |
| Year 1999 | 26 |  | 2.22 |  |
| Year 2006 | 45 |  | 2.03 |  |
| Year 2011 | 19 |  | 1.99 |  |
| Sussman (1991) | 41 |  | 2.21 |  |
| Simmen et al. (2010) |  |  |  |  |
| Year 2006 | 8 |  | 2.35 |  |
| Year 2005 | 1 |  | 2.32 |  |
| Rainwater et al. (2009) | 40 |  | 2.15 |  |
|  |  |  |  |  |
|  |  | *unknown sex* | | |
| Larsen et al. (2011) | 27 |  | 2.37 |  |
| Miller et al. (2007) | 70 | 1.50 | 2.20 | 2.60 |
| Miller et al. (2007) | 19 | 1.15 | 1.99 | 2.45 |
| Dutton et al. (2003) | 19 | 1.15 | 1.99 | 2.45 |
|  |  |  |  |  |

**Table S1.12** Body mass records (in kg) for adult, free-ranging Coquerel`s sifaka (*Propithecus coquereli*) in natural habitats

| **Source** | **n** | **minimum** | **average** | **maximum** |
| --- | --- | --- | --- | --- |
|  |  | *females* | | |
| Andriantompohavana et al. (2006) | 9 |  | 3.70 |  |
| Bauchot and Stephan (1966) | 3 |  | 3.18 |  |
| Ravosa et al. (1993) | 5 |  | 4.28 |  |
| Gordon et al. (2013) | 28 |  |  | 4.05 |
|  |  |  |  |  |
|  |  | *males* | | |
| Andriantompohavana et al. (2006) | 16 |  | 3.70 |  |
| Bauchot and Stephan (1966) | 2 |  | 3.58 |  |
| Ravosa et al. (1993) | 10 |  | 3.70 |  |
| Gordon et al. (2013) | 44 |  |  | 4.17 |
|  |  |  |  |  |

**Table S1.13** Body mass records (in kg) for adult, free-ranging red ruffed lemur (*Varecia rubra*) in natural habitats

| **Source** | **n** | **minimum** | **average** | **maximum** |
| --- | --- | --- | --- | --- |
|  |  | *females* | | |
| Vasey (2007) | 2 |  | 3.64 |  |
|  |  |  |  |  |
|  |  | *males* | | |
| Vasey (2007) | 1 |  | 3.30 |  |
|  |  |  |  |  |
|  |  | *unknown sex* | | |
| Dutton et al. (2008) | 22 | 2.08 | 2.95 | 3.60 |
|  |  |  |  |  |

**Table S1.14** Body mass records (in kg) for adult, free-ranging black-and-white ruffed lemur (*Varecia variegata*) in natural habitats

| **Source** | **n** | **minimum** | **average** | **maximum** |
| --- | --- | --- | --- | --- |
|  |  | *females* | | |
| Baden et al. (2008) | 47 |  | 3.70 |  |
| Junge and Baden (2021) | 41 |  | 3.40 |  |
| Morland (1991) | 7 |  | 3.07 |  |
| Smith and Jungers (1997) | 5 |  | 3.52 |  |
|  |  |  |  |  |
|  |  | *males* | | |
| Bauchot and Stephan (1966) | 2 |  | 2.97 |  |
| Baden et al. (2008) | 51 |  | 3.60 |  |
| Junge and Baden (2021) | 39 |  | 3.26 |  |
| Morland (1991) | 2 |  | 3.25 |  |
| Smith and Jungers (1997) | 13 |  | 3.63 |  |
|  |  |  |  |  |
|  |  | *unknown sex* | | |
| Terranova and Coffman (1997) | 18 | 3.00 | 3.60 | 4.10 |
|  |  |  |  |  |

**Table S2.1** Body mass records (in kg) for adult, free-ranging Angolan colobus (*Colobus angolensis*) in natural habitats

| **Source** | **n** | **minimum** | **average** | **maximum** |
| --- | --- | --- | --- | --- |
|  |  | *females* | | |
| Colyn (1994) | 5 | 6.40 | 7.77 | 9.15 |
| Oates (1994) | 6 |  | 7.40 |  |
| Napier and Napier (1985) | 5 |  | 7.40 |  |
| Delson (2000)  *C. angolensis* | 2 | 6.40 | 10.10 | 9.15 |
| *C. a. palliates* | 1 |  | 9.10 |  |
|  |  |  |  |  |
|  |  | *males* | | |
| Colyn (1994) | 8 | 7.60 | 9.62 | 12.6 |
| Oates (1994) | 4 |  | 9.80 |  |
| Napier and Napier (1985) | 2 |  | 9.68 |  |
| Delson (2000)  *C. angolensis* | 2 | 7.60 | 10.10 | 12.6 |
| *C. a. palliates* | 3 | 8.73 | 9.44 | 10.59 |
|  |  |  |  |  |

**Table S2.2** Body mass records (in kg) for adult, free-ranging manteled guereza (*Colobus guereza*) in natural habitats

| **Source** | **n** | **minimum** | **average** | **maximum** |
| --- | --- | --- | --- | --- |
|  |  | *females* | | |
| Jungers (1985) | 10 |  | 8.04 |  |
| Napier and Napier (1985) | 12 |  | 7.83 |  |
| Oates (1994)  *C. g. guereza* | 4 |  | 9.20 |  |
| *C. g. matshiei* | 7 |  | 7.90 |  |
| Bauchot and Stephan (1969) | 2 | 5.7 | 7.00 | 8.3 |
| Ruff (2003) | 9 |  | 7.93 |  |
| Delson (2000)  *C. g. guereza* | 3 | 8.20 | 9.23 | 10.00 |
| *C. g gallarum* | 3 | 6.10 | 7.60 | 8.70 |
| *C. g kikuyuensis* | 1 |  | 9.03 |  |
| *C. g matschiei* | 18 | 6.42 | 7.89 | 10.23 |
| *C. g occidentalis* | 46 | 5.44 | 7.42 | 10.88 |
|  |  |  |  |  |
|  |  | *males* | | |
| Jungers (1985) | 10 |  | 10.10 |  |
| Napier and Napier (1985) | 8 |  | 9.33 |  |
| Oates (1994)  *C. g. guereza* | 3 |  | 13.50 |  |
| *C. g. matschiei* | 4 |  | 10.10 |  |
| Bauchot and Stephan (1969) | 1 |  | 4.00 |  |
| Ruff (2003) | 9 |  | 9.49 |  |
| Delson (2000)  *C. g. guereza* | 6 | 9.75 | 11.87 | 14.40 |
| *C.g dodingae* | 1 |  | 10.43 |  |
| *C. g gallarum* | 1 |  | 9.40 |  |
| *C. g kikuyuensis* | 3 | 7.10 | 9.84 | 11.41 |
| *C. g matschiei* | 20 | 8.00 | 9.75 | 11.79 |
| *C. g occidentalis* | 46 | 6.80 | 9.08 | 11.34 |
|  |  |  |  |  |

**Table S2.3** Body mass records (in kg) for adult, free-ranging king colobus (*Colobus polykomos*) in natural habitats

| **Source** | **n** | **minimum** | **average** | **maximum** |
| --- | --- | --- | --- | --- |
|  |  | *females* | | |
| Napier and Napier (1985) | 6 |  | 7.13 |  |
| Oates et al. (1990) | 10 | 6.60 | 8.30 | 10.00 |
| Bauchot and Stephan (1969) | 5 |  | 8.79 |  |
| Delson (2000)  *C. polykomos* | 16 | 6.20 | 7.93 | 10.00 |
| *C. p. dollmani* | 2 | 6.10 | 7.30 | 8.50 |
| *C. p. vellerosus* | 5 | 6.20 | 6.86 | 7.50 |
|  |  |  |  |  |
|  |  | *Males* | | |
| Napier and Napier (1985) | 5 |  | 8.42 |  |
| Oates et al. (1990) | 5 | 8.00 | 9.90 | 11.70 |
| Bauchot and Stephan (1969) | 1 |  | 10.00 |  |
| Delson (2000)  *C. polykomos* | 8 | 8.00 | 10.18 | 11.70 |
| *C. p. dollmani* | 2 | 9.10 | 9.55 | 10.00 |
| *C. p. vellerosus* | 3 | 8.00 | 8.50 | 9.10 |
|  |  |  |  |  |

**Table S2.4** Body mass records (in kg) for adult, free-ranging northern plains gray langur (*Semnopithecus entellus*) in natural habitats

| **Source** | **n** | **minimum** | **average** | **maximum** |
| --- | --- | --- | --- | --- |
|  |  | *females* | | |
| Bishop (1975), Blaffer Hrdy (1977) | 12 |  | 11.20 |  |
| Bishop (1975)  *S. e. achilles* | 2 |  | 16.10 |  |
| Mohonot pers.com., cited in Roonwal (1981) | 8 |  | 13.12 |  |
| Oboussier&Maydell (1960), cited in Roonwal (1981)  *S. entellus* | 2 |  | 10.25 |  |
| *S. e. schistacea* | 5 |  | 15.78 |  |
| *S. e. achates* | 2 |  | 7.00 |  |
| Pocock (1928a), cited in Roonwal (1981) |  |  |  |  |
| *S. entellus* | 1 |  | 11.30 |  |
| *S. e. schistacea* | 2 |  | 15.90 |  |
| *S. e. achates* | 3 |  | 10.03 |  |
| *S. e. thersites* | 8 |  | 6.84 |  |
| Pocock (1928b), cited in Roonwal (1981)  *S. e. anchises* | 2 |  | 10.65 |  |
| *S. e. priam* | 1 |  | 8.80 |  |
| Pocock (1939), cited in Roonwal (1981)  *S. e. anchises* | 2 |  | 10.65 |  |
| *S. e. priam* | 1 |  | 10.65 |  |
| Shortrige (1914), cited in Roonwal (1981) | 1 |  | 12.20 |  |
| Delson (2000)  *S. entellus* | 1 |  | 11.34 |  |
| *S. e. achates* | 3 | 7.71 | 10.05 | 12.24 |
| *S. e. aeneas* | 1 |  | 9.97 |  |
| *S. e. ajax* | 1 |  | 12.70 |  |
| *S. e. elissa* | 2 | 8.278 | 9.35 | 10.43 |
| *S. e. hector* | 2 | 13.154 | 13.60 | 14.06 |
| *S. e. iulus* | 1 |  | 8.39 |  |
| *S. e. priam* | 1 |  | 8.84 |  |
| *S. e. schistacea* | 3 | 11.34 | 14.82 | 17.23 |
| *S. e. thersites* | 5 | 5.45 | 6.99 | 8.61 |
|  |  |  |  |  |
|  |  |  |  |  |
|  |  |  |  |  |
|  |  | *males* | | |
| Bishop (1975) |  |  |  |  |
| *S. entellus* | 4 |  | 18.20 |  |
| *S. e. achilles* | 2 |  | 17.00 |  |
| Delson (2000) |  |  |  |  |
| *S. entellus* | 1 |  | 15.87 |  |
| *S. e. achates* | 3 | 10.31 | 13.26 | 15.87 |
| *S. e. aeneas* | 1 |  | 11.56 |  |
| *S. e. ajax* | 2 | 19.50 | 19.95 | 20.41 |
| *S. e. hector* | 1 |  | 17.23 |  |
| *S. e. iulus* | 1 |  | 9.52 |  |
| *S. e. priam* | 1 |  | 16.78 |  |
| *S. e. schistacea* | 1 |  | 23.58 |  |
| *S. e. thersites* | 5 | 7.93 | 11.23 | 13.38 |
| Oboussier&Maydell (1960), cited in Roonwal (1981) |  |  |  |  |
| *S. entellus* | 3 |  | 9.40 |  |
| *S. e. schistacea* | 2 |  | 18.00 |  |
| *S. e. achates* | 2 |  | 9.5 |  |
| Phillips (1935), cited in Roonwal (1981) | 6 |  | 12.50 | 13.40 |
| Pocock (1928a), cited in Roonwal (1981) |  |  |  |  |
| *S. entellus* | 1 |  | 15.90 |  |
| *S. e. ajax* | 1 |  | 19.05 |  |
| *S. e. schistacea* | 1 |  | 17.20 |  |
| *S. e. achates* | 4 |  | 12.12 |  |
| *S. e. iulus* | 1 |  | 9.50 |  |
| *S. e. aeneas* | 1 |  | 11.60 |  |
| *S. e. priam* | 1 |  | 16.80 |  |
| *S. e. thersites* | 2 |  | 12.35 |  |
| Pocock (1939), cited in Roonwal (1981) |  |  |  |  |
| *S. e. ajax* | 1 |  | 20.90 |  |
| *S. e. achilles* | 1 |  | 15.90 |  |
| Shortrige (1914), cited in Roonwal (1981) | 1 |  | 15.90 |  |
| ZSI Coll., cited in Roonwal (1981) |  |  |  |  |
| *S. e. thersites* | 1 |  | 9.20 |  |
|  |  |  |  |  |

**Table S2.5** Body mass records (in kg) for adult, free-ranging east javan langur (*Trachypithecus auratus*) in natural habitats

| **Source** | **n** | **minimum** | **average** | **maximum** |
| --- | --- | --- | --- | --- |
|  |  | *males* | | |
| Kool (1993) | - |  | 7.10 |  |
|  |  |  |  |  |
|  |  | *unknown sex* | | |
| Fleagle (1999), cited in Leca et al. (2013) | - |  | 6.20 |  |
|  |  |  |  |  |

**Table S2.6** Body mass records (in kg) for adult, free-ranging silvery langur (*Trachypithecus cristatus*) in natural habitats

| **Source** | **n** | **minimum** | **average** | **maximum** |
| --- | --- | --- | --- | --- |
|  |  | *females* | | |
| Oates (1994) | 25 |  | 5.70 |  |
| Wright and Willis (2012) | - |  | 8.93 |  |
| Ruff (2002) | 5 |  | 5.80 |  |
| Fooden (1976) | 4 | 4.55 | 5.30 | 5.25 |
| Delson (2000)  *T. cristatus* | 15 | 4.99 | 6.13 | 7.59 |
| *T. c. ultima* | 26 | 4.99 | 5.67 | 6.35 |
|  |  |  |  |  |
|  |  | *males* | | |
| Oates (1994) | 13 |  | 6.60 |  |
| Wright and Willis (2012) | - |  | 6.61 |  |
| Ruff (2002) | 5 |  | 7.10 |  |
| Fooden (1976) | 1 |  | 7.10 |  |
| Delson (2000)  *T. cristatus* | 7 | 6.12 | 7.22 | 8.16 |
| *T. c. ultima* | 18 | 4.99 | 6.32 | 7.93 |
| *T. c. germaini* | 1 |  | 8.62 |  |
|  |  |  |  |  |

**Table S2.7** Body mass records (in kg) for adult, free-ranging Francois’ langur (*Trachypithecus francoisi*) in natural habitats

| **Source** | **n** | **minimum** | **average** | **maximum** |
| --- | --- | --- | --- | --- |
|  |  | *females* | | |
| Delson (2000) | 3 | 7.20 | 7.80 | 8.70 |
| Wright and Willis (2012) | - |  | 6.17 |  |
|  |  |  |  |  |
|  |  | *males* | | |
| Delson (2000) | 8 | 5.70 | 7.96 | 9.45 |
| Wright and Willis (2012) | - |  | 6.50 |  |
|  |  |  |  |  |

**Table S2.8** Body mass records (in kg) for adult, free-ranging dusky leaf monkey (*Trachypithecus obscurus*) in natural habitats

| **Source** | **n** | **minimum** | **average** | **maximum** |
| --- | --- | --- | --- | --- |
|  |  | *females* | | |
| Burton (1981) | 36 | 4.95 | 6.47 | 7.89 |
| Napier and Napier (1985) | 22 |  | 6.60 |  |
| Fooden (1976) | 12 | 4.12 | 6.03 | 7.25 |
| Fleagle (1976), cited in Plavcan and Van Schaik (1997) | 2 |  | 6.60 |  |
| Harves et al. (1987), cited in Plavcan and Van Schaik (1997) | 1 |  | 6.50 |  |
| Lyon Jr (1907) | 1 |  | 6.12 |  |
| Delson (2000)  *T. obscurus* | 11 | 4.99 | 6.79 | 8.84 |
| *T. o. carbo* | 1 |  | 5.44 |  |
| *T. o. flavicauda* | 7 | 4.99 | 6.86 | 8.61 |
| *T. o. sanctorum* | 1 |  | 8.16 |  |
| *T. o. subsp Indet.* | 4 | 4.15 | 5.12 | 6.25 |
|  |  |  |  |  |
|  |  | *males* | | |
| Burton (1981) | 15 | 6.30 | 7.39 | 9.00 |
| Napier and Napier (1985) | 12 |  | 7.32 |  |
| Fooden (1976) | 12 | 6.5 | 7.60 | 8.3 |
| Fleagle (1976), cited in Plavcan and Van Schaik (1997) | 2 |  | 7.58 |  |
| Harves et al. (1987), cited in Plavcan and Van Schaik (1997) | 1 |  | 8.30 |  |
| Lyon Jr (1907) | 1 |  | 6.57 |  |
| Delson (2000)  *T. obscurus* | 13 | 6.80 | 8.03 | 9.18 |
| *T. o. carbo* | 2 | 8.165 | 8.33 | 8.50 |
| *T. o. flavicauda* | 2 | 7.03 | 7.25 | 7.48 |
| *T. o. sanctorum* | 1 |  | 10.88 |  |
| *T. o. subsp Indet.* | 3 | 6.10 | 7.30 | 8.70 |
|  |  |  |  |  |

**Table S2.9** Body mass records (in kg) for adult, free-ranging proboscis monkey (*Nasalis larvatus*) in natural habitats

| **Source** | **n** | **minimum** | **average** | **maximum** |
| --- | --- | --- | --- | --- |
|  |  | *females* | | |
| Schultz (1941) | 15 | 8.16 | 9.83 | 11.79 |
| Lyon Jr (1907) | 3 |  | 9.03 |  |
| Lyon Jr (1907) | 3 |  | 9.22 |  |
| Oates (1994) | 14 |  | 10.00 |  |
| Ruff (2003) | 5 |  | 10.56 |  |
| Delson (2000) | 24 | 7.25 | 9.63 | 11.79 |
| Wright and Willis (2012) | - |  | 6.19 |  |
|  |  |  |  |  |
|  |  | *males* | | |
| Schultz (1941) | 10 |  | 20.34 |  |
| Lyon Jr (1907) | 3 |  | 20.00 |  |
| Lyon Jr (1907) | 6 |  | 20.63 |  |
| Oates (1994) | 13 |  | 21.20 |  |
| Ruff (2003) | 6 |  | 20.95 |  |
| Delson (2000) | 28 | 13.26 | 19.75 | 23.58 |
| Wright and Willis (2012) | - |  | 19.89 |  |
| Koda, Murai et al. (2018) | 18 |  | 21.64 |  |
|  |  |  |  |  |

**Table S2.10** Body mass records (in kg) for adult, free-ranging red-shanked douc (*Pygathrix nemaeus*) in natural habitats

| **Source** | **n** | **minimum** | **average** | **maximum** |
| --- | --- | --- | --- | --- |
|  |  | *females* | | |
| Willis (1995) | 2 |  | 8.44 |  |
| Delson (2000)  *P. nemaeus* | 1 |  | 8.16 |  |
| *P. n. nigripes* | 1 |  | 8.70 |  |
|  |  |  |  |  |
|  |  | *males* | | |
| Willis (1995) | 4 |  | 11.00 |  |
| Delson (2000)  *P. nemaeus* | 2 | 10.43 | 10.88 | 11.34 |
| *P. n. nigripes* | 2 | 11.00 | 11.05 | 11.10 |
|  |  |  |  |  |

**Figure S1a** Gompertz growth models (green line) fitted to the age-specific body mass data of females and males of zoo-kept lemur species. For a description of the model parameters, see Table S1.

**Lemurs**

| *Eulemur albifrons* |  |
| --- | --- |
|  |  |
| *Eulemur collaris* |  |
|  |  |
| *Eulemur coronatus* |  |
|  |  |
| *Eulemur flavifrons* |  |
|  |  |
| *Eulemur fulvus* |  |
|  |  |
| *Eulemur macaco* |  |
|  |  |
| *Eulemur mongoz* |  |
|  |  |
|  |  |
| *Eulemur rubriventer* |  |
|  |  |
| *Eulemur rufus* |  |
|  |  |
| *Hapalemur alaotrensis* |  |
|  |  |

| *Lemur catta* |  |
| --- | --- |
|  |  |
| *Propithecus coquereli* |  |
|  |  |
| *Varecia rubra* |  |
|  |  |
| *Varecia variegata* |  |
|  |  |

**Figure S1b** Gompertz growth models (green line) fitted to the age-specific body mass data of females and males of zoo-kept colobine monkey species. For a description of the model parameters, see Table S1.

**Colobine monkeys**

| *Colobus angolensis* |  |
| --- | --- |
|  |  |
| *Colobus guereza* |  |
|  |  |
| *Colobus polykomos* |  |
|  |  |
| *Semnopithecus entellus* |  |
|  |  |
| *Trachypithecus auratus* |  |
|  |  |

| *Trachypithecus cristatus* |  |
| --- | --- |
|  |  |
| *Trachypithecus francoisi* |  |
|  |  |
| *Trachypithecus obscurus* |  |
|  |  |
| *Nasalis larvatus* |  |
|  |  |
| *Pygathrix nemaeus* |  |
|  |  |

**Table S3a** Results of Gompertz model fit (according to $y=A {e^{-e}}^{-k\left( t-t_{0} \right)}$) to the age-specific body mass data of females and males of lemur species kept in zoos, and the resulting threshold age for defining adulthood. *A* = asymptotic adult body mass; *k* = relative growth rate; *t_0_* = time until maximum growth.

| **Species** | **Sex** | **Asymptote mass**  **(*A*; kg)** | **Time to maximum growth**  **(*t_0_*, years)** | **Relative growth rate**  **(*k*; d^-1^)** | **Threshold age (years)** |
| --- | --- | --- | --- | --- | --- |
|  |  |  |  |  |  |
| *Eulemur albifrons* | Female | 2.13 ±0.01 | 3.50 ±2.80 | 3.44 ±1.29 | 1.2 |
|  | Male | 2.22 ±0.02 | 1.69 ±0.85 | 1.60 ±0.58 | 2.2 |
|  |  |  |  |  |  |
| *Eulemur collaris* | Female | 2.59 ±0.01 | 1.19 ±0.04 | 0.95 ±0.03 | 3.3 |
|  | Male | 2.36 ±0.00 | 1.36 ±0.07 | 1.52 ±0.06 | 2.2 |
|  |  |  |  |  |  |
| *Eulemur coronatus* | Female | 1.63 ±0.00 | 1.61 ±0.07 | 1.62 ±0.06 | 2.1 |
|  | Male | 1.64 ±0.00 | 1.67 ±0.05 | 1.62 ±0.04 | 2.1 |
|  |  |  |  |  |  |
| *Eulemur flavifrons* | Female | 2.59 ±0.01 | 2.20 ±0.07 | 1.61 ±0.05 | 2.3 |
|  | Male | 2.51 ±0.01 | 2.12 ±0.07 | 1.54 ±0.05 | 2.4 |
|  |  |  |  |  |  |
| *Eulemur fulvus* | Female | 2.54 ±0.01 | 2.17 ±0.21 | 1.79 ±0.17 | 2.1 |
|  | Male | 2.46 ±0.01 | 2.12 ±0.12 | 2.03 ±0.13 | 1.8 |
|  |  |  |  |  |  |
| *Eulemur macaco* | Female | 2.48 ±0.01 | 2.01 ±0.10 | 1.60 ±0.08 | 2.3 |
|  | Male | 2.46 ±0.01 | 1.87 ±0.10 | 1.65 ±0.09 | 2.2 |
|  |  |  |  |  |  |
| *Eulemur mongoz* | Female | 1.63 ±0.00 | 1.87 ±0.07 | 1.70 ±0.06 | 2.1 |
|  | Male | 1.58 ±0.00 | 2.18 ±0.06 | 2.00 ±0.05 | 1.9 |
|  |  |  |  |  |  |
| *Eulemur rubriventer* | Female | 2.07 ±0.01 | 0.84 ±0.03 | 0.55 ±0.02 | 5.1 |
|  | Male | 2.02 ±0.00 | 0.78 ±0.02 | 0.57 ±0.01 | 4.8 |
|  |  |  |  |  |  |
| *Eulemur rufus* | Female | 2.29 ±0.01 | 1.29 ±0.13 | 1.32 ±0.13 | 2.4 |
|  | Male | 2.35 ±0.01 | 1.61 ±0.13 | 1.46 ±0.11 | 2.4 |
|  |  |  |  |  |  |
| *Hapalemur alaotrensis* | Female | 1.43 ±0.01 | 1.92 ±0.09 | 1.43 ±0.06 | 2.5 |
|  | Male | 1.45 ±0.00 | 2.22 ±0.07 | 1.86 ±0.05 | 2.0 |
|  |  |  |  |  |  |
| *Lemur catta* | Female | 2.81 ±0.00 | 1.79 ±0.02 | 1.14 ±0.01 | 3.1 |
|  | Male | 2.92 ±0.00 | 1.88 ±0.02 | 1.21 ±0.01 | 3.0 |
|  |  |  |  |  |  |
| *Propithecus coquereli* | Female | 4.25 ±0.01 | 2.73 ±0.04 | 1.52 ±0.02 | 2.6 |
|  | Male | 3.77 ±0.01 | 2.79 ±0.03 | 1.74 ±0.02 | 2.3 |
|  |  |  |  |  |  |
| *Varecia rubra* | Female | 3.82 ±0.00 | 2.30 ±0.04 | 2.38 ±0.04 | 1.6 |
|  | Male | 3.61 ±0.00 | 2.49 ±0.04 | 2.76 ±0.04 | 1.4 |
|  |  |  |  |  |  |
| *Varecia variegata* | Female | 3.94 ±0.00 | 2.29 ±0.03 | 2.07 ±0.03 | 1.8 |
|  | Male | 3.64 ±0.00 | 2.33 ±0.02 | 2.49 ±0.02 | 1.5 |
|  |  |  |  |  |  |

**Table S3b** Results of Gompertz model fit (according to $y=A {e^{-e}}^{-k\left( t-t_{0} \right)}$) to the age-specific body mass data of females and males of colobine monkey species kept in zoos, and the resulting threshold age for defining adulthood. *A* = asymptotic adult body mass; *k* = relative growth rate; *t_0_* = time until maximum growth.

| **Species** | **Sex** | **Asymptote mass**  **(*A*; kg)** | **Time to maximum growth**  **(*t_0_*, years)** | **Relative growth rate**  **(*k*; d^-1^)** | **Threshold age (years)** |
| --- | --- | --- | --- | --- | --- |
|  |  |  |  |  |  |
| *Colobus angolensis* | Female | 10.61 ±0.03 | 2.50 ±0.07 | 0.60 ±0.01 | 6.4 |
|  | Male | 12.56 ±0.04 | 3.17 ±0.12 | 0.68 ±0.02 | 6.0 |
|  |  |  |  |  |  |
| *Colobus guereza* | Female | 11.30 ±0.02 | 2.37 ±0.03 | 0.54 ±0.01 | 7.1 |
|  | Male | 13.06 ±0.02 | 2.65 ±0.03 | 0.60 ±0.01 | 6.6 |
|  |  |  |  |  |  |
| *Colobus polykomos* | Female | 11.07 ±0.10 | 2.76 ±0.09 | 0.61 ±0.03 | 6.5 |
|  | Male | 15.95 ±0.29 | 2.00 ±0.13 | 0.31 ±0.02 | 11.7 |
|  |  |  |  |  |  |
| *Semnopithecus entellus* | Female | 11.91 ±0.20 | 5.50 ±2.52 | 0.93 ±0.18 | 5.0 |
|  | Male | 17.39 ±0.31 | 3.48 ±0.77 | 0.57 ±0.07 | 7.5 |
|  |  |  |  |  |  |
| *Trachypithecus auratus* | Female | 9.63 ±0.08 | 2.87 ±0.19 | 0.53 ±0.03 | 7.6 |
|  | Male | 8.73 ±0.06 | 3.68 ±0.31 | 0.82 ±0.04 | 5.2 |
|  |  |  |  |  |  |
| *Trachypithecus cristatus* | Female | 7.63 ±0.04 | 2.60 ±0.11 | 0.63 ±0.02 | 6.2 |
|  | Male | 8.01 ±0.04 | 2.46 ±0.09 | 0.72 ±0.02 | 5.4 |
|  |  |  |  |  |  |
| *Trachypithecus francoisi* | Female | 7.60 ±0.02 | 2.47 ±0.04 | 0.66 ±0.01 | 5.9 |
|  | Male | 8.26 ±0.02 | 2.69 ±0.04 | 0.66 ±0.01 | 6.0 |
|  |  |  |  |  |  |
| *Trachypithecus obscurus* | Female | 7.33 ±0.02 | 2.96 ±0.09 | 0.73 ±0.02 | 5.6 |
|  | Male | 7.44 ±0.03 | 3.03 ±0.13 | 0.87 ±0.03 | 4.7 |
|  |  |  |  |  |  |
| *Nasalis larvatus* | Female | 9.63 ±0.10 | 2.66 ±0.61 | 0.45 ±0.06 | 8.8 |
|  | Male | 21.03 ±0.81 | 3.27 ±0.66 | 0.30 ±0.05 | 14.0 |
|  |  |  |  |  |  |
| *Pygathrix nemaeus* | Female | 11.43 ±0.02 | 3.21 ±0.06 | 0.59 ±0.01 | 7.0 |
|  | Male | 15.49 ±0.04 | 2.98 ±0.05 | 0.49 ±0.01 | 8.2 |

**Figure S2** Example for the visual display of body mass development in a ringtailed lemur (*Lemur catta*). Note a general trend of body mass decline with age, an irregular change of body mass in cycles spanning several years, and several instances of a seasonal (annual) fluctuation. Crosses represent the average body mass in winter (blue) and summer (red) periods. Trend line in black dashes.

**Table S4** Body mass records (in kg) and patterns for adult, zoo-kept lemur species of the present study. Note that this selection of individuals is based on data availability and not necessarily representative for the current global zoo populations.

| **Species** | **Sex** | **n** | **mean ±SD**  **(min, max)** | **% seasonal fluctuations (of n)** | **% old age decline (of n)** |
| --- | --- | --- | --- | --- | --- |
| *Eulemur albifrons* | Female | 40 | 2.20±0.28  (1.65, 3.00) | 100%  (3) | 100%  (3) |
|  | Male | 49 | 2.29 ±0.44  (1.50, 3.63) | 57%  (7) | 100%  (6) |
|  |  |  |  |  |  |
| *Eulemur collaris* | Female | 42 | 2.47 ±0.32^A^  (1.77, 3.20) | 50%  (16) | 81%  (16) |
|  | Male | 49 | 2.35 ±0.20^B^  (1.90, 2.86) | 84%  (19) | 60%  (20) |
|  |  |  |  |  |  |
| *Eulemur coronatus* | Female | 83 | 1.61 ±0.18  (1.16, 1.99) | 76%  (17) | 67%  (18) |
|  | Male | 111 | 1.65 ±0.21  (1.05, 2.20) | 83%  (18) | 45%  (20) |
|  |  |  |  |  |  |
| *Eulemur flavifrons* | Female | 53 | 2.53 ±0.31^A^  (1.70, 3.27) | 75%  (20) | 79%  (19) |
|  | Male | 62 | 2.41 ±0.28^B^  (1.92, 3.04) | 86%  (29) | 73%  (26) |
|  |  |  |  |  |  |
| *Eulemur*  *fulvus* | Female | 66 | 2.48 ±0.44  (1.73, 3.54) | 60%  (5) | 75%  (4) |
|  | Male | 67 | 2.47 ±0.36  (1.84, 3.72) | 82%  (11) | 63%  (8) |
|  |  |  |  |  |  |
| *Eulemur macaco* | Female | 126 | 2.61 ±0.47  (1.59, 3.78) | 65%  (17) | 59%  (17) |
|  | Male | 140 | 2.54 ±0.41  (1.81, 3.96) | 86%  (22) | 67%  (21) |
|  |  |  |  |  |  |
| *Eulemur mongoz* | Female | 84 | 1.63 ±0.24^a^  (1.13, 2.34) | 75%  (28) | 63%  (27) |
|  | Male | 93 | 1.57 ±0.24^b^  (1.17, 2.54) | 68%  (38) | 47%  (36) |
|  |  |  |  |  |  |
| *Eulemur rubiventer* | Female | 74 | 2.08 ±0.29  (1.63, 3.00) | 65%  (23) | 79%  (19) |
|  | Male | 72 | 2.03 ±0.24  (1.41, 2.88) | 60%  (15) | 69%  (13) |
|  |  |  |  |  |  |
| *Eulemur*  *rufus* | Female | 46 | 2.30 ±0.35  (1.65, 3.33) | 90%  (10) | 64%  (11) |
|  | Male | 52 | 2.30 ±0.31  (1.60, 3.05) | 100%  (15) | 54%  (13) |
|  |  |  |  |  |  |
| *Hapalemur alaotrensis* | Female | 59 | 1.38 ±0.27  (0.92, 2.35) | 100%  (12) | 100%  (13) |
|  | Male | 74 | 1.40 ±0.23  (0.93, 2.07) | 81%  (16) | 100%  (17) |
|  |  |  |  |  |  |
| *Lemur*  *catta* | Female | 1624 | 2.76 ±0.46^a^  (1.31, 4.57) | 90%  (180) | 59%  (190) |
|  | Male | 1858 | 2.87 ±0.48^b^  (1.50, 4.99) | 87%  (227) | 82%  (223) |
|  |  |  |  |  |  |
| *Propithecus coquereli* | Female | 56 | 4.17 ±0.48^a^  (2.87, 5.85) | 100%  (20) | 94%  (18) |
|  | Male | 61 | 3.76 ±0.36^b^  (2.97, 4.65) | 61%  (18) | 100%  (16) |
|  |  |  |  |  |  |
| *Varecia*  *rubra* | Female | 396 | 3.67±0.55^a^  (2.20, 5.59) | 91%  (78) | 60%  (82) |
|  | Male | 515 | 3.54 ±0.53^b^  (2.06, 6.00) | 95%  (111) | 76%  (92) |
|  |  |  |  |  |  |
| *Varecia variegata* | Female | 527 | 3.77 ±0.59^a^  (2.08, 6.00) | 95%  (94) | 78%  (80) |
|  | Male | 708 | 3.57 ±0.51^b^  (2.23, 5.59) | 90%  (132) | 76%  (84) |
|  |  |  |  |  |  |

AB, ab indicate significant sexual dimorphism, as assessed by parametric t-test (A,B) or nonparametric U-test (a,b) in the case of not normally distributed data.

**Table S5** Body mass records (in kg) and patterns for adult, zoo-kept colobine monkey species in the present study. Note that this selection of individuals is based on data availability and not necessarily representative for the current global zoo populations.

| **Species** | **Sex** | **n** | **mean ±SD**  **(min, max)** | **% seasonal fluctuations (of n)** | **% old age decline (of n)** |
| --- | --- | --- | --- | --- | --- |
| *Colobus angolensis* | Female | 68 | 10.17 ±1.78^A^  (6.82, 15.12) | 20%  (10) | 75%  (4) |
|  | Male | 54 | 11.71 ±1.75^B^  (8.72, 15.24) | 0%  (7) | 100%  (2) |
|  |  |  |  |  |  |
| *Colobus guereza* | Female | 346 | 10.39±1.98^a^  (6.47, 17.86) | 5%  (15) | 83%  (18) |
|  | Male | 301 | 12.65 ±2.11^b^  (7.40, 20.50) | 6%  (33) | 86%  (21) |
|  |  |  |  |  |  |
| *Colobus polykomos* | Female | 9 | 10.89 ±1.61  (8.00, 13.05) | 0%  (1) | 0%  (1) |
|  | Male | 6 | 12.75 ±2.78  (9.28, 17.19) | 0%  (1) | 100%  (1) |
|  |  |  |  |  |  |
| *Semnopithecus entellus* | Female | 48 | 12.47 ±3.02^A^  (5.09, 20.00) | 0%  (1) | 100%  (2) |
|  | Male | 17 | 17.50 ±3.74^B^  (10.50, 25.00) | 0%  (1) | -  - |
|  |  |  |  |  |  |
| *Trachypithecus auratus* | Female | 71 | 7.62 ±1.44^a^  (5.13, 13.53) | 0%  (2) | 50%  (2) |
|  | Male | 39 | 8.35 ±1.21^b^  (6.00, 10.73) | 0%  (2) | 100%  (1) |
|  |  |  |  |  |  |
| *Trachypithecus cristatus* | Female | 57 | 6.80 ±1.70^a^  (3.81, 11.37) | 0%  (6) | 100%  (5) |
|  | Male | 55 | 7.89 ±1.55^b^  (5.58, 11.73) | 40%  (5) | 75%  (4) |
|  |  |  |  |  |  |
| *Trachypithecus francoisi* | Female | 92 | 7.17 ±1.01^a^  (4.85, 11.01) | 7%  (14) | 73%  (11) |
|  | Male | 105 | 7.97 ±0.70^b^  (6.45, 10.32) | 6%  (18) | 61%  (18) |
|  |  |  |  |  |  |
| *Trachypithecus obscurus* | Female | 60 | 6.76 ±1.06^a^  (4.00, 8.89) | 0%  (6) | 86%  (7) |
|  | Male | 51 | 7.44 ±1.09^b^  (5.79, 11.00) | 20%  (5) | 100%  (7) |
|  |  |  |  |  |  |
| *Nasalis larvatus* | Female | 13 | 9.37 ±1.08^A^  (7.03, 11.29) | -  - | -  - |
|  | Male | 5 | 17.63 ±5.53^B^  (10.21, 26.46) | -  - | -  - |
|  |  |  |  |  |  |
| *Pygathrix nemaeus* | Female | 27 | 8.05±1.92^a^  (5.30, 13.64) | 0%  (4) | 100%  (4) |
|  | Male | 19 | 11.08 ±2.69^b^  (8.75, 17.99) | 67%  (3) | 67%  (3) |
|  |  |  |  |  |  |

AB, ab indicate significant sexual dimorphism, as assessed by parametric t-test (A,B) or nonparametric U-test (a,b) in the case of not normally distributed data.

**Figure S3a** Comparison of body masses of zoo lemurs (black dots) as compared to the body mass range of adult individuals in natural habitats (blue shading)

*Eulemur albifrons*

*Eulemur collaris*

*Eulemur coronatus*

*Eulemur flavifrons*

*Eulemur fulvus*

*Eulemur macaco*

*Eulemur mongoz*

*Eulemur rubriventer*

*Eulemur rufus*

*Hapalemur alaotrensis*

*Lemur catta*

*Propithecus coquereli*

*Varecia rubra*

*Varecia variegata*

**Figure S3b** Comparison of body masses of zoo colobine monkeys (black dots) as compared to the body mass range of adult individuals in natural habitats (blue shading)

*Colobus angolensis*

*Colobus guereza*

*Colobus polykomos*

*Semnopithecus entellus*

*Trachypithecus auratus*

*Trachypithecus cristatus*

*Trachypithecus francoisi*

*Trachypithecus obscurus*

*Nasalis larvatus*

*Pygathrix nemaeus*

**Figure S4a** Classification of individual zoo lemurs (black dots) in comparison to the mean wild body mass (stipled line) into ‘underweight’ (blue), ‘healthy’ (green), ‘overweight (yellow), ‘obese’ (orange) and ‘very obese’ (red)

**Figure S4b** Classification of individual zoo colobine monkeys (black dots) in comparison to the mean wild body mass (stipled line) into ‘underweight’ (blue), ‘healthy’ (green), ‘overweight (yellow), ‘obese’ (orange) and ‘very obese’ (red)

**Figure S5a** Historical development in lemurs of the proportion of ‘underweight’ (blue), ‘healthy’ (green), ‘overweight (yellow), ‘obese’ (orange) and ‘very obese’ (red) individuals from the year on in which at least data for 10 individuals were available

**Figure S5b** Historical development in colobine monkeys of the proportion of ‘underweight’ (blue), ‘healthy’ (green), ‘overweight (yellow), ‘obese’ (orange) and ‘very obese’ (red) individuals from the year on in which at least data for 10 individuals were available

**References**

Andriantompohavana, R., Zaonarivelo, J. R., Randriamampionona, R., Razafindraibe, J. F. X., Brenneman, R., & Louis Jr, E. E. (2006). A preliminary study on resident lemur populations in the Mariarano Classified Forest. *Lemur News, 11*, 21-24.

Baden, A. L., Brenneman, R. A., & Louis Jr, E. E. (2008). Morphometrics of wild black‐and‐white ruffed lemurs (*Varecia variegata*). *American Journal of Primatology, 70*, 913-926.

Bauchot, R., & Stephan, H. (1966). Données nouvelles sur l’encéphalisation des insectivores et des prosimiens. *Mammalia, 30*, 160-196.

Bauchot, R., & Stephan, H. (1969). Encéphalisation et niveau évolutif chez les simiens. *Mammalia, 33*, 225-275.

Bayart, F., & Simmen, B. (2005). Demography, range use, and behavior in black lemurs (*Eulemur macaco macaco*) at Ampasikely, northwest Madagascar. *American Journal of Primatology, 67*, 299-312.

Bishop, N. H. (1975). *Social behavior of langur monkeys (presbytis entellus) in a high altitude environment*. PhD thesis, University of California, Berkeley.

Blaffer Hrdy, S. (1977). *The Langurs of Abu. Female and Male Strategies of Reproduction*. Harvard University Press, Cambridge MA.

Bradley, B., Stumpf, R., & Wright, P. (1997). Morphometrics of *Eulemur fulvus* *albocollaris* in Vevembe Forest, Madagascar. *American Journal of Physical Anthropology, 24*, 79-80.

Colyn, M. (1994). Données pondérales sur les primates Cercopithecidae d’Afrique Centrale (Bassin du Zaïre/Congo). *Mammalia, 58*, 483-488.

Delson, E. (2000). Body mass in Cercopithecidae (Primates, Mammalia): estimation and scaling in extinct and extant taxa. *American Museum of Natural History, Anthropological Papers, 83*, 1-159.

Donati, G., Kesch, K., Ndremifidy, K., Schmidt, S., Ramanamanjato, J.-B., Borgognini-Tarli, S. M., & Ganzhorn, J. U. (2011). Better few than hungry: flexible feeding ecology of collared lemurs *Eulemur collaris* in littoral forest fragments. *PLoS One, 6*, e19807.

Dutton, C. J., Junge, R. E., & Louis, E. E. (2003). Biomedical evaluation of free-ranging ring-tailed lemurs (*Lemur catta*) in Tsimanampetsotsa strict nature reserve, Madagascar. *Journal of Zoo and Wildlife Medicine, 34*, 16-24.

Dutton, C. J., Junge, R. E., & Louis, E. E. (2008). Biomedical evaluation of free-ranging red ruffed lemurs (*Varecia rubra*) within the Masoala National Park, Madagascar. *Journal of Zoo and Wildlife Medicine, 39*, 76-85.

Gerson, J. (1999). *Size in Eulemur fulvus rufus from western Madagascar: Sexual dimorphism and ecogeographic variation.* *American Journal of Physical Anthropology, Suppl. 28*, 134.

Glander, K. E., Wright, P. C., Daniels, P. S., & Merenlender, A. M. (1992). Morphometrics and testicle size of rain forest lemur species from southeastern Madagascar. *Journal of Human Evolution, 22*, 1-17.

Gordon, A. D., Johnson, S. E., & Louis, E. E. (2016). Environmental correlates of body mass in true lemurs (*Eulemur* spp.). *International Journal of Primatology, 37*, 89-108.

Gordon, A. D., Johnson, S. E., & Louis Jr, E. E. (2013). Females are the ecological sex: Sex‐specific body mass ecogeography in wild sifaka populations (*Propithecus* spp.). *American Journal of Physical Anthropology, 151*, 77-87.

Ichino, S., Chatani, K., Kawamoto, Y., Sato, H., Schnöll, A., Soma, T., Koyama, N., Aimi, M., & Takahata, Y. (2013). Decrease in the body mass of wild ringtailed lemurs at Berenty Reserve in Madagascar with environmental changes. *African Study Monographs, 34*, 109-118.

Johnson, S. E., Gordon, A. D., Stumpf, R. M., Overdorff, D. J., & Wright, P. C. (2005). Morphological variation in populations of *Eulemur albocollaris* and *E. fulvus rufus*. *International Journal of Primatology, 26*, 1399-1416.

Junge, R. E., & Baden, A. L. (2021). Health assessment of black-and-white ruffed lemurs (*varecia variegata*) in Ranomafana National Park, Madagascar. *Journal of Zoo and Wildlife Medicine, 52*, 1095-1104.

Junge, R. E., Dutton, C. J., Knightly, F., Williams, C. V., Rasambainarivo, F. T., & Louis, E. E. (2008). Comparison of biomedical evaluation for white-fronted brown lemurs (*Eulemur fulvus albifrons*) from four sites in Madagascar. *Journal of Zoo and Wildlife Medicine, 39*, 567-575.

Junge, R. E., & Louis, E. E. (2005). Biomedical evaluation of two sympatric lemur species (*Propithecus verreauxi deckeni* and *Eulemur fulvus rufus*) in Tsiombokibo Classified Forest, Madagascar. *Journal of Zoo and Wildlife Medicine, 36*, 581-589.

Junge, R. E., & Louis, E. E. (2007). Biomedical evaluation of black lemurs (*Eulemur macaco macaco*) in Lokobe Reserve, Madagascar. *Journal of Zoo and Wildlife Medicine, 38*, 67-76.

Jungers, W. L. (1985). Body size and scaling of limb proportions in primates. In Jungers, W. L. (ed.) *Size and Scaling in Primate Biology*. Springer, Boston MA, pp. 345-381.

Kool, K. M. (1993). The diet and feeding behavior of the Silver leaf monkey (*Trachypithecus auratus sondaicus*) in Indonesia. *International Journal of Primatology, 14*, 667-700.

Koyama, N., Aimi, M., Kawamoto, Y., Hirai, H., Go, Y., Ichino, S., & Takahata, Y. (2008). Body mass of wild ring-tailed lemurs in Berenty Reserve, Madagascar, with reference to tick infestation: a preliminary analysis. *Primates, 49*, 9-15.

Larsen, R. S., Moresco, A., Sauther, M. L., & Cuozzo, F. P. (2011). Field anesthesia of wild ring-tailed lemurs (*Lemur catta*) using tiletamine–zolazepam, medetomidine, and butorphanol. *Journal of Zoo and Wildlife Medicine, 42*, 75-87.

Leca, J.-B., Gunst, N., Rompis, A., Soma, G., Putra, I. A., & Wandia, I. N. (2013). Population density and abundance of ebony leaf monkeys (*Trachypithecus auratus*) in West Bali National Park, Indonesia. *Primate Conservation, 26*, 133-144.

Miller, D. S., Sauther, M. L., Hunter-Ishikawa, M., Fish, K., Culbertson, H., Cuozzo, F. P., Campbell, T. W., Andrews, G. A., Chavey, P. S., Nachreiner, R., Rumbeiha, W., Stacewicz-Sapuntzakis, M., & Lappin, M. R. (2007). Biomedical evaluation of free-ranging ring-tailed lemurs (*Lemur catta*) in three habitats at the Beza Mahafaly Special Reserve, Madagascar. *Journal of Zoo and Wildlife Medicine, 38*, 201-216.

Morland, H. S. (1991). Social organization and ecology of black and white ruffed lemurs (*Varecia variegata variegata*) in lowland rain forest, Nosy Mangabe, Madagascar. PhD thesis, Yale University.

Mutschler, T., Nievergelt, C. M., & Feistner, A. T. (2000). Social organization of the Alaotran gentle lemur (*Hapalemur griseus alaotrensis*). *American Journal of Primatology, 50*, 9-24.

Napier, P. H., & Napier, J. R. (1985). *The Natural History of the Primates*. MIT Press, Cambridge, MA.

Oates, J. F. (1994). The natural history of African colobines. In: Oates, J. F. & Davies, A. G. (eds.) *Colobine Monkeys: Their Ecology, Behaviour and Evolution*. Cambridge University Press, Cambridge MA, pp. 75-128.

Oates, J. F., Whitesides, G. H., Davies, A. G., Waterman, P. G., Green, S. M., Dasilva, G. L., & Mole, S. (1990). Determinants of variation in tropical forest primate biomass: new evidence from West Africa. *Ecology, 71*, 328-343.

Rabarivola, C., Prosper, P., Zaramody, A., Andriaholinirina, N., & Hauwy, M. (2007). Cytogenetics and taxonomy of the genus *Hapalemur*. *Lemur News, 12*, 26-29.

Rainwater, T. R., Sauther, M. L., Rainwater, K. A. E., Mills, R. E., Cuozzo, F. P., Zhang, B., McDaniel, L. N., Abel, M. T., Marsland, E. J., Weber, M. A., Jacky, I. A. Y., Platt, S. G., Cobb, G. P., & A, A. T. (2009). Assessment of organochlorine pesticides and metals in ring‐tailed lemurs (*Lemur catta*) at Beza Mahafaly Special Reserve, Madagascar. *American Journal of Primatology, 71*, 998-1010.

Randriatahina, G. H., & Roeder, J.-J. (2012). Group size, composition and stability in a wild population of blue-eyed black lemurs (*Eulemur flavifrons*) at Ankarafa, Sahamalaza National Park. In: Tuttle, R. (ed.) *Leaping Ahead: Advances in Prosimian Biology*. Springer, New York, pp. 127-136.

Rasmussen, M. A. (2005). Seasonality in predation risk: Varying activity periods in lemurs and other primates. In: Brockman, D. K. & van Schaik, C. P. (eds.) *Seasonality in Primates: Studies of Living and Extinct Human and Nonhuman Primates.* Cambridge University Press, Cambridge MA, pp. 105-128.

Ravosa, M. J., Meyers, D. M., & Glander, K. E. (1993). Relative growth of the limbs and trunk in sifakas: heterochronic, ecological, and functional considerations. *American Journal of Physical Anthropology, 92*, 499-520.

Roonwal, M. (1981). Intraspecific variation in size, proportion of body parts and weight in the Hanuman langur, *Presbytis entellus*, in south Asia, with remarks on subspeciation. *Records of the Zoological Survey of India, 79*, 125-158.

Ruff, C. B. (2003). Long bone articular and diaphyseal structure in Old World monkeys and apes. II: Estimation of body mass. *American Journal of Physical Anthropology, 120*, 16-37.

Sato, H. (2013). Habitat shifting by the common brown lemur (*Eulemur fulvus fulvus*): a response to food scarcity. *Primates, 54*, 229-235.

Sauther, M. L., Fish, K. D., Cuozzo, F. P., Miller, D. S., Hunter-Ishikawa, M., & Culbertson, H. (2006). Patterns of health, disease, and behavior among wild ringtailed lemurs, *Lemur catta*: effects of habitat and sex. In: Jolly, A., Sussman, R. W., Koyama, N. & Rasamimanana, H.(eds.) *Ringtailed Lemur Biology: Lemur catta in Madagascar*. Springer, Boston MA, pp. 313-331.

Simmen, B., Bayart, F., Rasamimanana, H., Zahariev, A., Blanc, S., & Pasquet, P. (2010). Total energy expenditure and body composition in two free-living sympatric lemurs. *PLoS One, 5*, e9860.

Smith, R. J., & Jungers, W. L. (1997). Body mass in comparative primatology. *Journal of Human Evolution, 32*, 523-559.

Steffens, K. J. E., Sanamo, J., & Razafitsalama, J. (2022). The role of lemur seed dispersal in restoring degraded forest ecosystems in Madagascar. *Folia Primatologica, 93*, 1-19.

Sussman, R. W. (1991). Demography and social organization of free‐ranging *Lemur catta* in the Beza Mahafaly Reserve, Madagascar. *American Journal of Physical Anthropology, 84*, 43-58.

Terranova, C. J., & Coffman, B. S. (1997). Body weights of wild and captive lemurs. *Zoo Biology, 16*, 17-30.

Vasey, N. (2007). The breeding system of wild red ruffed lemurs (*Varecia rubra*): a preliminary report. *Primates, 48*, 41-54.

Zaonarivelo, J. R., Andriantompohavana, R., Razafindrakoto, A., Andrianasolo, J., Rajaobelina, S., Brenneman, R. A., Wohlhauser, S., & Louis Jr, E. E. (2007). Preliminary lemur survey of Andakoera classified forest an regional forest fragments od Madagascar *Lemur News, 12*, 26-29.
